# Using linear and natural cubic splines, SITAR, and latent trajectory models to characterise nonlinear longitudinal growth trajectories in cohort studies

**DOI:** 10.1186/s12874-022-01542-8

**Published:** 2022-03-15

**Authors:** Ahmed Elhakeem, Rachael A. Hughes, Kate Tilling, Diana L. Cousminer, Stefan A. Jackowski, Tim J. Cole, Alex S. F. Kwong, Zheyuan Li, Struan F. A. Grant, Adam D. G. Baxter-Jones, Babette S. Zemel, Deborah A. Lawlor

**Affiliations:** 1 MRC Integrative Epidemiology Unit at the University of Bristol, Bristol, UK; 2 Population Health Sciences, Bristol Medical School, University of Bristol, Bristol, UK; 3 Division of Human Genetics, Children’s Hospital of Philadelphia, Philadelphia, PA USA; 4 Department of Genetics, University of Pennsylvania, Philadelphia, PA USA; 5 Center for Spatial and Functional Genomics, Children’s Hospital of Philadelphia, Philadelphia, PA USA; 6 College of Kinesiology, University of Saskatchewan, Saskatoon, Saskatchewan Canada; 7 Children’s Hospital of Eastern Ontario Research Institute, Ottawa, Ontario Canada; 8 UCL Great Ormond Street Institute of Child Health, London, UK; 9 Division of Psychiatry, Centre for Clinical Brain Sciences, University of Edinburgh, Edinburgh, UK; 10 School of Mathematics and Statistics, Henan University, Kaifeng, Henan China; 11 Department of Statistics and Actuarial Sciences, Simon Fraser University, Burnaby, BC Canada; 12 Department of Pediatrics, University of Pennsylvania Perelman School of Medicine, Philadelphia, PA USA; 13 Division of Endocrinology and Diabetes, Children’s Hospital of Philadelphia, Philadelphia, PA USA; 14 Division of Gastroenterology, Hepatology and Nutrition, Children’s Hospital of Philadelphia, Philadelphia, PA USA

**Keywords:** ALSPAC, Bone mineral content, BMDCS, Growth models, Life-course, Mixed-effects, PBMAS, Tutorial

## Abstract

**Background:**

Longitudinal data analysis can improve our understanding of the influences on health trajectories across the life-course. There are a variety of statistical models which can be used, and their fitting and interpretation can be complex, particularly where there is a nonlinear trajectory. Our aim was to provide an accessible guide along with applied examples to using four sophisticated modelling procedures for describing nonlinear growth trajectories.

**Methods:**

This expository paper provides an illustrative guide to summarising nonlinear growth trajectories for repeatedly measured continuous outcomes using (i) linear spline and (ii) natural cubic spline linear mixed-effects (LME) models, (iii) Super Imposition by Translation and Rotation (SITAR) nonlinear mixed effects models, and (iv) latent trajectory models. The underlying model for each approach, their similarities and differences, and their advantages and disadvantages are described. Their application and correct interpretation of their results is illustrated by analysing repeated bone mass measures to characterise bone growth patterns and their sex differences in three cohort studies from the UK, USA, and Canada comprising 8500 individuals and 37,000 measurements from ages 5–40 years. Recommendations for choosing a modelling approach are provided along with a discussion and signposting on further modelling extensions for analysing trajectory exposures and outcomes, and multiple cohorts.

**Results:**

Linear and natural cubic spline LME models and SITAR provided similar summary of the mean bone growth trajectory and growth velocity, and the sex differences in growth patterns. Growth velocity (in grams/year) peaked during adolescence, and peaked earlier in females than males e.g., mean age at peak bone mineral content accrual from multicohort SITAR models was 12.2 years in females and 13.9 years in males. Latent trajectory models (with trajectory shapes estimated using a natural cubic spline) identified up to four subgroups of individuals with distinct trajectories throughout adolescence.

**Conclusions:**

LME models with linear and natural cubic splines, SITAR, and latent trajectory models are useful for describing nonlinear growth trajectories, and these methods can be adapted for other complex traits. Choice of method depends on the research aims, complexity of the trajectory, and available data. Scripts and synthetic datasets are provided for readers to replicate trajectory modelling and visualisation using the R statistical computing software.

**Supplementary Information:**

The online version contains supplementary material available at 10.1186/s12874-022-01542-8.

## Background

Appropriate modelling of repeated measures in cohort studies can improve our understanding of: (i) patterns of change across the life-course (e.g., developmental trajectories to peak function and age-related decline); (ii) influences on these patterns of change; and (iii) influence of variation in patterns of change on later health and wellbeing [1]. Many developmental processes display non-linear patterns of change with age, especially during the growing years, which makes it important but challenging to accurately model their trajectories [2]. Also requiring attention is the choice of method to appropriately address the research question, e.g., whether to use methods that model an average trajectory in the whole sample [3–5] or clustering based approaches to identify groups of individuals with similar trajectories [6]. Moreover, there is a lack of accessible and practical guidance which can discourage novice researchers from using more complex methods [7]. Thus, an overview of sophisticated modelling procedures along with open-source software applications and application in multiple different cohorts can help address these challenges.

This paper provides a guide to describing nonlinear longitudinal growth trajectories for a single repeatedly-measured continuous outcome using linear and natural cubic regression splines [3, 4], SITAR (Super Imposition by Translation and Rotation) models [5], and (spline-based) latent trajectory models [6] – all common methods for examining growth. The next section of the paper gives an overview of modelling nonlinear growth and the various models considered. The four approaches (and the appropriate interpretation of their results) are then illustrated by modelling bone mass trajectories across three cohort studies to characterise patterns of change and their sex differences. The final section provides recommendations about when different modelling methods might be useful and discusses approaches and challenges in analysing exposures and outcomes of patterns of change, and in making cross-cohort comparisons.

## Methods

### Modelling nonlinear growth

A variety of statistical methods are available for handling repeated (correlated) observations from the same individuals and analysing trajectories [8]. Most methods involve fitting growth models within either a structural equation modelling framework (e.g., latent growth curve analysis where change is analysed as a latent process [9]) or a multilevel modelling framework, with both giving similar results under certain conditions [10]. One type of repeated measures model that is useful when the primary interest is estimating a population-average trajectory is generalised estimating equations (GEE) [11, 12]. This uses a working covariance matrix to correct for the dependence among repeated observations but is usually not suited for examining variation within/between individuals. Another repeated measures model that can estimate both population-average and individual-specific trajectories (and is more robust to missing outcome data than GEE) is the mixed-effects model [13].

### Mixed-effects models

Mixed-effects models (random-effects, multilevel, or hierarchical models) estimate a population-average trajectory as ‘fixed effects’ and variation of individual trajectories around this average as ‘random effects’ [12–14]. A common form is the linear mixed-effects (LME) model where the repeated outcome is modelled by a linear combination of the fixed and random effects. An LME model for a single continuous outcome (e.g., weight), as a *linear* function of time, which includes random intercepts and random slopes can be written as follows:

1 $${y}_{ij}={\beta}_{oi}+{\beta}_{1i}{t}_{ij}+{\varepsilon}_{ij},{\varepsilon}_{ij}\sim N\left(0,{\sigma}_{\varepsilon}^2\right),i.i.d.$$

1.1 $${\beta}_{0i}={\beta}_0+{u}_{0i},{u}_{0i}\sim N\left(0,{\sigma}_0^2\right),i.i.d.$$

1.2 $${\beta}_{1i}={\beta}_1+{u}_{1i},{u}_{1i}\sim N\left(0,{\sigma}_1^2\right),i.i.d.$$

where, *y*_*ij*_ denotes a single outcome *y* measured in individual *i* (*i* = 1, 2, …, *N*) at time *t*_*ij*_ (*j* = 1, 2, …, *J*_*i*_), with responses (*y*_1_…*y*_*N*_) assumed to be independent between individuals. *β*_*oi*_ and *β*_1*i*_ are individual-specific intercept and slope terms (respectively) that have fixed effects (*β*_*o*_, *β*_1_) and random effects (*u*_*oi*_, *u*_1*i*_). The random effects *u*_*i*_ are assumed to be independently normally distributed with mean zero and covariance matrix *Ω*_*u*_. Residual errors *ε*_*ij*_ are assumed to be independently identically normally distributed with variance ${\sigma}_{\varepsilon}^2$ and reflect the difference between observed and predicted values for individual *i* at occasion *j*. The random effects *u*_*i*_ and residuals *ε*_*ij*_ are assumed to be mutually independent.

### Moving beyond a linear trajectory

**Fig. 1:**
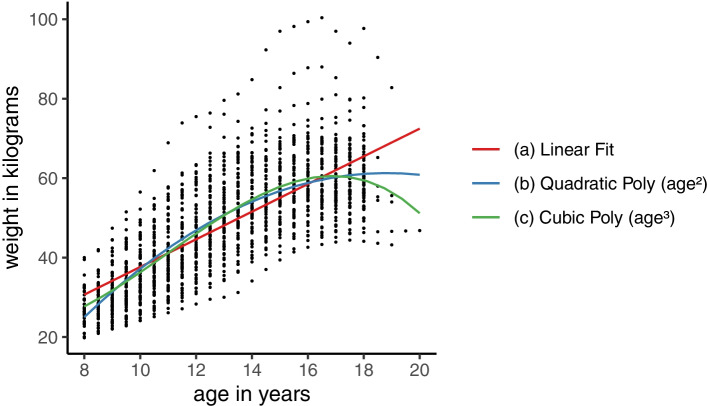
Example illustrating the limitations of using polynomial functions to approximate a nonlinear growth trajectory. Coloured lines represent predicted trajectories from LME models with age as (**a**) linear term and as (**b**) quadratic polynomial and (**c**) cubic polynomial. Points display weight measurements taken from 70 females in the Berkeley Child Guidance Study. Dataset was originally provided as an appendix to the book by Tuddenham and Snyder (1954). The data for this example were taken from the freely accessible ‘Berkeley’ dataset provided with the *‘sitar’* package [15]

The LME model in Eq. (1) assumes linear change in the outcome with increasing time (e.g., age). Nonlinear change, which is common (particularly when modelling change over a large age range and/or during periods of rapid complex growth) can be incorporated into LME models by including linear combinations of nonlinear terms for age in the model – i.e., keeping the linear link function. Historically, the standard approach has been to make use of polynomial functions to approximate nonlinear curves. However, as illustrated in Fig. 1, polynomials have limitations, e.g., simpler polynomials give few curve shapes and more complex polynomials tend to fit badly at extremes and produce artefactual turns in the curve. A more flexible alternative to analysing complex patterns of change (e.g., with several peaks/troughs, as with body mass index (BMI) over infancy, childhood, and adolescence) is using a set of connected polynomials covering different segments of the time/age distribution known as spline functions.

### Spline functions

A spline function is a set of piecewise polynomials that are joined together at break/turning points called knots (see [16] for an introductory review). Splines are formed using spline basis functions (the dimensional space containing each element from a set of local polynomials) and can be fitted by various methods including linear and nonlinear models. The polynomial degree and method of knot placement is generally what distinguishes between different spline functions. There are three main ways of estimating spline functions: as regression splines, penalised regression splines, and smoothing splines [12, 16–19]. Regression splines use fewer knots than observations – here both the number and location of knots must be specified by the user. Penalised regression splines are regression splines that include an added penalty on the parameters – here, the user does not need to specify the knots, instead, a (maximum) spline basis complexity (flexibility) is specified, and the spline coefficients are then penalised by a tuning parameter to avoid overfitting. Smoothing splines use a knot at every unique observation with penalisation of the estimated curve – these preceded penalised regression splines and are now less widely used.

In this paper, we consider two commonly used types of regression splines: linear splines and natural cubic splines. These can be parameterised by a linear combination of the transformed covariate (e.g., age) and thus they can be fitted within an LME framework to model a repeated outcome as a nonlinear function of time. Fitting penalised regression spline models is covered briefly in the Discussion section. The next three sections give an overview to using linear and natural cubic splines in LME models and approaches to choosing the number and position of knots. SITAR, which utilises a natural cubic spline within a nonlinear mixed effects framework, is then covered. These three mixed-effects modelling approaches all estimate population-average trajectories (and individual variation from these) and so can be used when it is hypothesised that the study population shares a common mean trajectory. Extensions to latent trajectory models (with trajectory shapes estimated using natural cubic splines) for situations where the aim/hypothesis is to identify suspected latent heterogenous trajectories in subpopulations as opposed to one mean trajectory for the whole population are covered in the subsequent section. All four modelling approaches are summarised in Table 1.

**Table 1:** Overview of linear spline LME models, natural cubic spline LME models, SITAR, and latent trajectory models for analysing nonlinear growth trajectories of a single repeatedly measured continuous outcome

	Linear spline LME model	Natural cubic spline LME model	SITAR	Latent trajectory model
Description	linear mixed-effects model with a linear spline function of the independent time variable	linear mixed-effects model with a restricted cubic spline function of the independent time variable	nonlinear mixed-effects model based on the shape invariant growth model	heterogenous growth curves fit to unknown subgroups of individuals
Advantages	easy to interpret the spline slope coefficients; can describe growth rate during different periods of the growth process	continuous 1^st^ & 2^nd^ derivatives give smoother trajectory and can identify points of peaks/troughs; linearity constraint gives a more reliable trajectory shape as less erratic at the tails of distribution	has useful features of the natural cubic spline, easy to estimate individual growth features – most notably individual ages at peak growth velocity	can identify unobserved sub-groups of individuals sharing distinct growth trajectories if any exist
Limitations	biologically implausible sudden changes in velocity (i.e., at the knots); erratic at the tails; cannot identify points of velocity maxima/minima; position (and location) of knots important	coefficients difficult to interpret (so plotting is more useful); can be challenging to estimate the individual growth curves due to complex spline basis functions used by the statistical software	may not work well for complex growth patterns e.g., with multiple peaks and troughs or where the growth curve does not plateau in adulthood	difficult to identify the optimal number of sub-groups; may identify implausible subgroups; trajectories tend not to replicate in other cohorts
R package(s)	lme4; lspline	lme4; splines	sitar	lcmm, splines

All models can include all individuals with at least one observed outcome measure, with valid estimates obtained under the assumption of outcome data missing at random (MAR) depending on observed values of the outcome and/or covariates. Note all models assume no autocorrelation (in our example, there are wide enough gaps between measures to assume that here)

### Linear spline LME models

The simplest spline function is the linear spline which can be used to describe growth by a series of connected lines joined at knots, where the slope can change after each knot [3]. For example, a linear spline for age (measured from 5 to 40 years) with 2 knots at ages 10 and 15 years produces three different linear slopes of the repeated outcome measure (e.g., weight): 5 to ≤10, 10 to ≤15, and 15 to ≤40 years. The LME model in Eq. (1) can be rewritten to include a linear spline function for age *b*(*t*) with *K* knots *ξ*_1_ < *ξ*_2_ < …*ξ*_*K*_ as:

2 $${y}_{ij}={\beta}_{0i}+{\beta}_{1i}{t}_{ij}+{\sum}_{k=1}^K{b}_{ki}{\left({t}_{ij}-{\xi}_k\right)}_{+}+{\varepsilon}_{ij}$$

2.1 $${\left({t}_{ij}-{\xi}_k\right)}_{+}=\left\{\ \begin{array}{c}0\ {t}_{ij}<{\xi}_k\ \\ {}\left({t}_{ij}-{\xi}_k\right)\ {t}_{ij}\ge {\xi}_k\end{array}\right.$$

The model in Eq. (2) includes a linear spline in both the fixed and random effects (with *b*_*ki*_ having fixed effect *b*_*k*_ and random effect *v*_*ki*_) which allows for nonlinear mean and individual trajectories, respectively. The fixed and random splines are assumed to have the same knots in Eq. (2) however, it is possible to allow fewer knots in the random spline. If the aim was solely to model a nonlinear mean trajectory, then Eq. (2) can be simplified by replacing the random spline with a random line – here, *β*_*oi*_ and *β*_1*i*_ have similar interpretation as in Eq. (1), with random effect *v*_*ki*_ omitted (i.e., *b*_*ki*_ = *b*_*k*_). The rate of change of a linear spline (1st derivative) is not continuous at the knots. An alternative function that has continuous 1^st^ and 2^nd^ derivatives is the natural cubic spline.

### Natural cubic spline LME models

A natural cubic spline (also known as restricted cubic spline) is a set of cubic polynomials with continuity and slope constraints at each knot, and additional constraint of linearity at the extremes of the curve, typically before the first and after the last knot [12, 16, 20, 21]. This extra linearity constraint makes the trajectory less erratic at the ends of the age distribution and so more reliable than linear splines (and unrestricted cubic splines). The inclusion of cubic terms with continuity constraints means natural cubic spline models can be more parsimonious for complex shapes than a linear spline with many knots. An LME model that includes a natural cubic spline function *b*(*t*) with *K* knots *ξ*_1_ < *ξ*_2_ < …*ξ*_*K*_ (and a linearity constraint for values *t* < *ξ*_1_ and *t* > *ξ*_*K*_) can be written as:

3 $${y}_{ij}={\beta}_{0i}+{\beta}_{1i}{t}_{ij}+{\sum}_{k=1}^{K-2}{b}_{ki}{\left({t}_{ij}-{\xi}_k\right)}_{\ast}^3+{\varepsilon}_{ij}$$

3.1 $${\left({t}_{ij}-{\xi}_k\right)}_{\ast}^3={\left({t}_{ij}-{\xi}_k\right)}_{+}^3-{\left({t}_{ij}-{\xi}_{K-1}\right)}_{+}^3\frac{\xi_K-{\xi}_k}{\xi_K-{\xi}_{K-1}}+{\left({t}_{ij}-{\xi}_K\right)}_{+}^3\frac{\xi_{K-1}-{\xi}_k}{\xi_K-{\xi}_{K-1}},k=1,2,\dots, K-2$$

In words, a natural cubic spline for age (measured from 5 to 40 years) with 2 knots at 10 and 15 years (and with first and last knots at 5 and 40 years, respectively), invokes 3 cubic polynomials: between 5 to ≤10, 10 to ≤15, and 15 to ≤40 years, and has its curvature equal to 0 at ages 5 and 40 years. If the first and last knots were placed at older and younger ages respectively, then the curve would be linear from the first and last knot to the youngest and oldest ages, respectively. Note, we have defined the natural cubic spline using truncated power basis (like how the linear spline was defined). The analysis software creates this function using a B-spline basis which is mathematically challenging to represent but numerically more stable. Whatever basis is used, if the polynomial degree and knots are identical, then the spline will always be the same.

### Choosing number and location of knots

The flexibility of regression splines is determined by the number/position of the knot points. For a natural cubic spline, the number of knots (rather than their position) is more important [20]. A small number of knots (between 3 and 5 knots) provides a good fit to some patterns [20] though, with many repeats (e.g., data spanning many decades) more knots may be required. Approaches to selecting the number/position of knots include (i) placing knots at quantiles of the age distribution (ii) using equally spaced knots, (iii) inspecting smoothing curves and using these to select knots, (iv) starting with many knots and reducing their number, and (v) placing knots at the mean age of data collection [3].

Model selection can be done informally (i.e., by inspecting plots of fitted values/residuals from competing models with different knots). Valid comparison between models with different knots (i.e., models with non-nested mean structures) can be done using likelihood-based information criteria (e.g., Bayesian Information Criterion value (BIC)) provided maximum likelihood (ML) estimation is used [22]. Note however, ML-based variance estimates are biased downwards [12] and so one approach is to compare models fitted by ML and by restricted maximum likelihood (REML). Cross-validation is also useful for model selection [23]. If knot position was a primary interest (e.g., testing sensitive periods), then topic knowledge can inform placement of knots [24].

### SITAR models

SITAR is a shape invariant nonlinear mixed-effects model [5, 25]. Whereas LME spline models are linear models that allow terms to describe a non-linear trajectory with age, nonlinear mixed-effects models are fundamentally nonlinear in the coefficients [12]. SITAR assumes that a study population has a common characteristic curve (fitted as fixed effects), which through shifting and scaling (by a set of 3 random effects) can be transformed into any individual curve. Following the notation in Cole et al [5, 26], a SITAR model for outcome *y* can be written as:

4 $${y}_{ij}={\alpha}_0+{\alpha}_i+h\left(\frac{t-{\beta}_0-{\beta}_i}{\exp \left(-{\gamma}_0-{\gamma}_i\right)}\right)+{\varepsilon}_{ij}$$

where *y*_*ij*_ is the outcome measurement for individual *i* at age *j*; *α*_0_, *β*_0_, *γ*_0_ are fixed effects; *α*_*i*_, *β*_*i*_, and *γ*_*i*_ are random effects for the *i* th individual; *h*(*t*) is a natural cubic spline curve; and *ε*_*ij*_ are independent normally distributed errors. The 3 random effects describe the size (*α*_*i*_), timing (*β*_*i*_), and intensity (*γ*_*i*_) of individual growth relative to the mean growth curve. *α*_*i*_ adjusts for the differences in *y* and geometrically reflects individual shifts up or down (translation) in the mean curve; *β*_*i*_ adjusts for differences in the timing of peak growth in *y* and geometrically reflects left to right shifts (translation) in the mean curve; and *γ*_*i*_ adjusts for the duration of the growth spurt and geometrically corresponds to shrinking or stretching of the age scale and rotating the curve.

Note, a key difference from other mixed effects models, with or without a natural cubic spline mean curve, is that SITAR models growth on both the x- and y-axes – this allows differences in developmental age to be modelled. Common practice in selecting the best fitting SITAR models is to compare models with varying number of knots placed at quantiles of the age distribution for the spline curve [5]. The internal SITAR model structure is also customisable [15].

### Latent trajectory models

The spline and SITAR models described above assume that the population is homogenous and described at the population level by a mean trajectory, with variability of individuals about this mean. As an alternative, latent trajectory models assume that there is a heterogenous population composed of unknown subgroups (latent classes) of individuals, each characterised by a unique mean trajectory profile [6, 27, 28]. Therefore, latent trajectory modelling addresses a different research hypothesis where heterogeneity in the population-average (mean) trajectory is of key interest. Latent trajectory models aim to minimise within group variance and maximise between group differences so that individuals are more similar within groups than between groups. Each individual has a probability of belonging to each latent class and is assigned to the class with the highest probability. Class membership is defined using a latent discrete random variable, with membership probability described by a multinomial logistic model.

Several latent trajectory modelling approaches are possible (see [6] for a recent overview). These include models that ignore the longitudinal structure (known as longitudinal latent class analysis) and models assuming no variability between individuals within subgroups (known as latent class growth analysis or group-based trajectory models). Another approach that is a direct extension of standard mixed-effects models is a growth mixture model, which involves fitting multiple growth curves to subgroups of individuals that share a common trajectory. Following the notation in [6], the LME model in Eq. (1) can be rewritten as a growth mixture model as:

5 $${y}_{ij\mid c}={\beta}_{0i}^c+{\beta}_{1i}^c{t}_{ij}+{\varepsilon}_{ij}^c\ \mathrm{for}\ c=1,\dots, C,$$

where *C* indicates number of latent classes, with probabilities *p*_*c*_, *c* = 1, …, *C*, with 0 ≤ *p*_*c*_ ≤ 1 and ${\sum}_{c=1}^C{p}_c=1$. All other terms are defined as before but specifically for each class *c*. Growth can be parameterised as nonlinear, e.g., using a natural cubic spline curve in each class (assuming same number/position of knots). Class-specific covariances for individual-level error terms can be included, and both fixed and random effects can be class specific.

Model estimation is conditional on a pre-specified number of classes, with the optimal number of classes identified through a combination of approaches. These include assessing interpretability and plausibility of classes e.g., inspecting if trajectories show biologically plausible patterns and examining characteristics (e.g., socioeconomic position) of the classes [29], information criteria, entropy (statistic for class separation), and numerically meaningful sub-groups (e.g., ≥5% class size). Models with > 1 class are prone to local maxima solutions (i.e., convergence to the best solution in a neighbourhood of the parameter space, rather than the global maximum (largest loglikelihood)). This can be avoided by using different starting values [30].

### Illustrative example

Next, using data from three cohort studies, we demonstrate the application of the four modelling approaches described above to characterise bone mineral content (BMC) growth trajectories and their sex differences, including in population subgroups with potentially distinct trajectories.

#### Bone mass through the life course

Bone mass in early life is thought to be an important determinant of fracture and osteoporosis risk in later life [31] however, few studies have described its developmental trajectory. Furthermore, sex differences in osteoporotic fracture are assumed to be due to menopause but may also reflect early sexual dimorphism in bone development. This is a timely exploration, given the availability of studies with repeated measurements of BMC (a marker of bone strength).

#### Studies and measurements

Three studies with repeated BMC measurements from childhood to adulthood were included (Table 2**,** Additional file 1): the Avon Longitudinal Study of Parents and Children (ALSPAC) [32, 33], Bone Mineral Density in Childhood Study (BMDCS) [34], and Pediatric Bone Mineral Accrual Study (PBMAS) [35]. Total-body (excluding-head) BMC was measured in grams using whole-body Dual-Energy X-ray Absorptiometry (DXA) scans. Of note, the studies used DXA devices from different manufacturers (Lunar vs. Hologic) which scale differently and are not interchangeable but repeat scans within studies were acquired on the same device. Individuals from each study were included if they had ≥1 measure of BMC and no missing data on age at DXA scan (in years) or sex. Analyses were restricted to white ethnicity because 2 cohorts were ethnically homogeneous [33, 35]. The final analysis samples comprised 3888 males and 4007 females in ALSPAC, 465 males and 488 females in BMDCS, and 112 males and 127 females in PBMAS. All studies had ethics approval and obtained parental or participant informed consent.

**Table 2:** Characteristics of the three cohort studies included in the trajectory modelling

Study name	Avon Longitudinal Study of Parents and Children (ALSPAC)	Bone Mineral Density in Childhood Study (BMDCS)	Pediatric Bone Mineral Accrual Study (PBMAS)
Design	birth cohort study (started in 1990–1992)	child cohort study (started in 2002–2003)	child cohort study (started in 1991)
Region and country	catchment area of 3 health authorities in Southwest England, UK	5 USA clinic centres: Los Angeles, New York, Cincinnati, Omaha, Philadelphia	2 elementary schools, Saskatoon, Saskatchewan, Canada
Birth years	1990–1992	1985–1997	1983–1976
Ethnicity	98% white ethnicity	ethnically diverse	95% white ethnicity
DXA device used to measure BMC	Lunar Prodigy	Hologic QDR-4500A	Hologic QDR-2000
Mean age at the baseline/youngest DXA scan (range)	9.9 years (8.8–11.7 years)	10.8 years (6.0–17.0 years)	11.8 years (8.0–15.1 years)
Mean age at last/oldest DXA scan (range)	24.6 years (22.4–26.5 years)	16.1 years (6.9–23.3 years)	37.3 years (34.3–40.2 years)
Frequency and the maximum number of repeated DXA scans	up to 6 repeated scans at mean ages 9.9, 11.7, 13.8, 15.4, 17.8, and 24.6 years	up to 7 yearly repeated scans	Up to 16 repeated scans (1991–1998, 2003–2005, 2007–2011 and 2016–2017)
Individuals included in the analysis^a^	4007 females 3888 males	488 females 465 males	127 females 112 males

^a^Trajectory modelling was restricted to white ethnicity individuals

#### Statistical analysis

Analyses were performed in R version 4.0.2 (R Project for Statistical Computing) and RStudio version 1.3.1 (RStudio Team). R code is available at https://github.com/aelhak/nltmr/. Synthetic versions of the PBMAS cohorts were simulated [36] and can be found in the same repository.

**Fig. 2:**
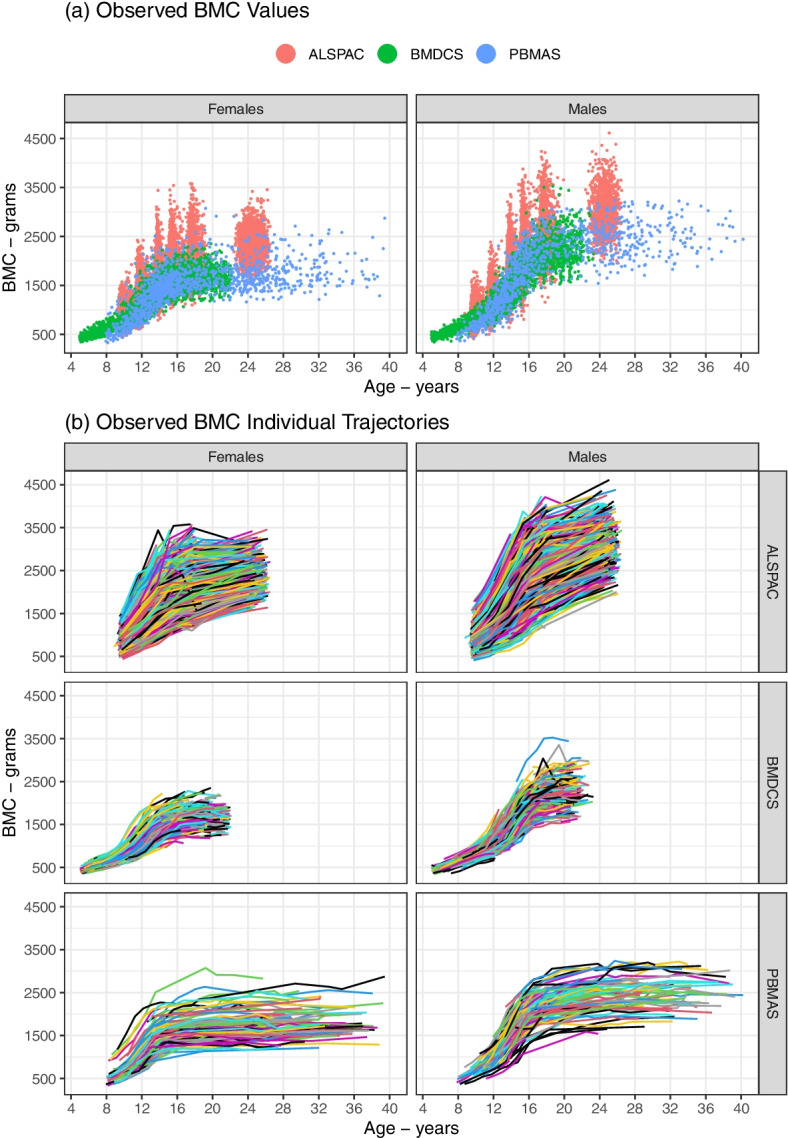
Plots of the cohort datasets used in the trajectory modelling showing (**a**) bone mineral content (BMC) values at each age, and (**b**) BMC individual trajectories. Figure shows the observed BMC values by cohort and sex (**a**), and the observed BMC individual trajectories by cohort and sex (**b**)

Prior to trajectory modelling, scatterplots of BMC against age, and line plots of the individual BMC trajectories were used to inspect the form of the trajectory and identify clearly outlying observations. The datasets used for trajectory modelling **(**Fig. 2**)** showed as expected nonlinear change in BMC with age, and higher BMC in ALSPAC due to Lunar device. The numbers of individuals at each visit were described and age and BMC were summarised with means and standard deviations (Additional file 1). Models were fitted separately by sex due to expected difference in bone mineral content accrual, and our aim was to explore this in the illustrative example.

Linear and natural cubic spline LME models were fitted using the ‘*lme4’* package [37]. Models with 2 to 6 knots (placed at quantiles of the age distribution) in the fixed effects curve were compared. Nonlinear individual trajectories were allowed by including a random effects spline with 1 knot at the median. SITAR models with 2 to 6 knots (at quantiles of the age distribution) in the spline curve (and three random-effects) were fitted using the ‘*sitar’* package [15]. LME and SITAR models were fitted by ML and best fitting models (optimum number of knots) were determined by the smallest BIC (Additional file 2). Goodness of fit for the selected models was assessed by examining residuals (conditional on the random effects) from LME models and variance in BMC explained by SITAR models. The selected models were used to describe BMC growth trajectory and growth velocity. Slopes of the fixed effects spline segments from the linear spline models were used to summarise mean BMC velocity during different age windows and identify windows for peak growth. Mean peak BMC velocity and age at peak BMC velocity were obtained from the natural cubic spline LME and SITAR models by differentiating the mean spline curves.

Growth mixture models were fitted using the ‘*lcmm’* package [30]. The forms of the best fitting mean natural cubic spline curves were used to model the fixed effects age curve. Models included random intercepts and random linear age slopes. Models with different numbers of latent classes were compared: from a 1-class model (i.e., a standard natural cubic spline LME model where all individuals follow a single mean trajectory) up to models with 5 classes. Models with > 1 class included class-specific random effects covariance matrices. An automatic search procedure was used to estimate each 2–5 class model for 100 iterations using random initial values from the distribution of the 1-class model. Optimal number of classes was chosen by inspecting predicted trajectory sub-groups from each model for biological plausibility, in addition to the smallest BIC and biggest entropy, and by excluding small class size (≥5%). Goodness of fit and discrimination capacity of the selected models (i.e. with optimal number of classes) was assessed by calculating posterior class membership probabilities [30].

## Results

**Fig. 3:**
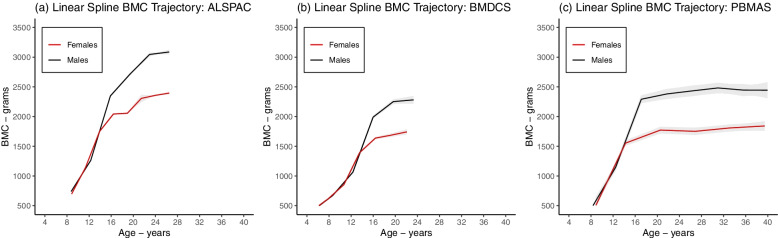
Mean BMC growth trajectory from the selected linear spline LME models. Figure shows the estimated mean BMC trajectory in females (black) and males (red) from linear spline LME models in ALSPAC (**a**), BMDCS (**b**), and PBMAS (**c**). Shaded areas around the mean trajectories represent 95% confidence intervals

**Fig. 4:**
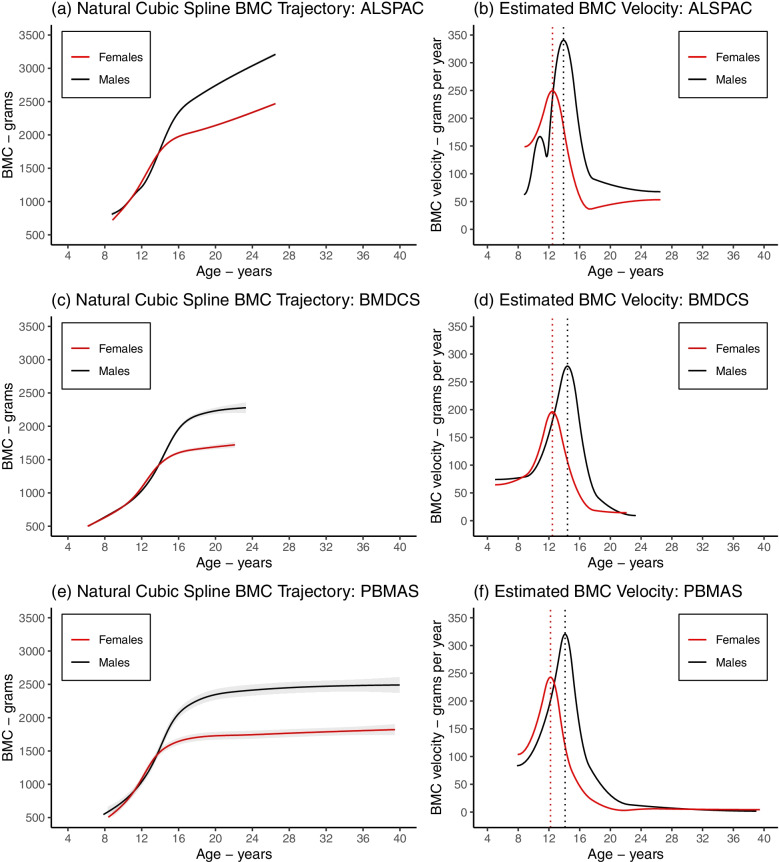
Mean BMC growth trajectory (left panels) and mean BMC growth velocity and age at peak velocity (right panels) from the selected natural cubic spline LME models. Figure shows the estimated mean BMC trajectory (**a**, **c**, **e**) and mean BMC growth velocity (**b**, **d**, **f**) in females (black) and males (red) from natural cubic spline LME models in ALSPAC (**a**, **b**), BMDCS (**c**, **d**), and PBMAS (**e**, **f**). The vertical lines in subplots b, d, f represent the mean age at peak velocity. Shaded areas around the mean growth trajectories represent 95% confidence intervals

**Fig. 5:**
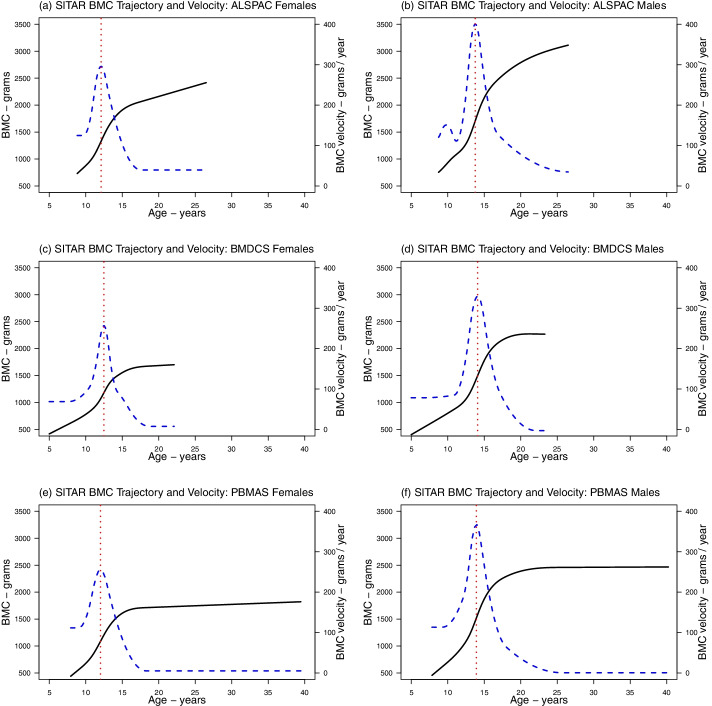
Mean BMC growth trajectory (solid black curves), mean BMC growth velocity (dashed blue curves), and mean age at peak BMC velocity (vertical red lines) from the selected SITAR models. Figure shows the estimated mean BMC trajectory (solid black curves), mean BMC growth velocity (dashed blue curves) and mean age at peak BMC velocity (vertical red lines) from SITAR models in ALSPAC females (**a**) and males (**b**), BMDCS females (**c**) and males (**d**), and PBMAS females (**e**) and males (**f**)

**Fig. 6:**
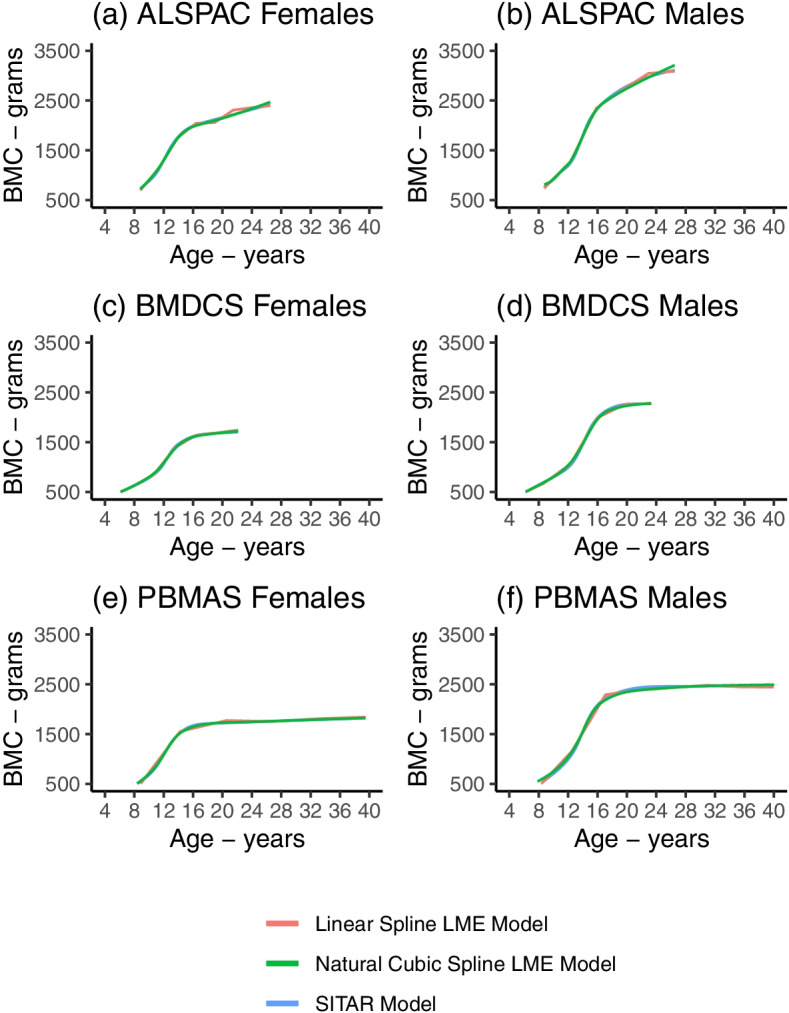
Overlayed mean BMC growth trajectories from the selected linear spline LME models, natural cubic spline LME models, and SITAR models. Figure shows the overlayed mean BMC growth trajectories from the selected linear spline LME models, natural cubic spline LME models, and SITAR models in ALSPAC females (**a**) and males (**b**), BMDCS females (**c**) and males (**d**), and PBMAS females (e) and males (**f**)

The mean predicted BMC trajectories in each cohort from the linear spline LME (Fig. 3), natural cubic spline LME (Fig. 4), and SITAR models (Fig. 5) showed that BMC increased with age up to a plateau in young adulthood, and thereafter remained stable to age 40 (in PBMAS). All models showed evidence of steeper growth trajectories through adolescence coinciding with emerging sex differences, with males subsequently having higher BMC than females, and plateauing later than females. BMC trajectories from all models were broadly similar (Fig. 6). Both linear and natural cubic spline LME models and SITAR provided a good fit to the data (**Online Resource 2**). The mean BMC growth velocity in different age windows (from linear spline LME models) peaked during adolescence, and the peak was lower and occurred earlier in females than males (Table 3). Mean BMC growth velocity curves from natural cubic spline LME and SITAR models were similar (Figs. 4-5): for all cohorts, the mean ages at peak BMC velocity from both models were within the age windows for peak growth identified by the linear spline LME models (Table 4).

**Table 3:** Estimated mean BMC growth velocity during different age windows from the selected linear spline LME models

	Grams per year change in BMC [(mean (95% CI)]		
	ALSPAC	BMDCS	PBMAS
*Females*			
8.8y to 11.4y (ALSPAC); 5.0y to 7.9y (BMDCS); 7.9y to 14.2y (PBMAS)	177.7 (173.8 to 181.6)	67.6 (60.1 to 75.1)	195.4 (189.7 to 201.1)
11.4y to 13.9y (ALSPAC); 7.9y to 10.7y (BMDCS); 14.2y to 20.5y (PBMAS)	238.9 (236.0 to 241.7)	84.1 (79.0 to 89.2)	34.7 (30.0 to 39.5)
13.9y to 16.4y (ALSPAC); 10.7y to 13.6y (BMDCS); 20.5y to 26.9y (PBMAS)	117.3 (113.2 to 121.3)	189.0 (184.7 to 193.3)	−3.3 (− 7.5 to 0.9)
16.4y to 18.9y (ALSPAC); 13.6y to 16.4y (BMDCS); 26.9y to 33.2y (PBMAS)	5.0 (−1.3 to 11.3)	85.7 (80.1 to 91.3)	9.1 (3.0 to 15.1)
18.9y to 21.5y (ALSPAC); 16.4y to 19.3y (BMDCS); 33.2y to 39.5y (PBMAS)	99.2 (74.2 to 124.2)	17.4 (10.6 to 24.1)	5.2 (−5.9 to 16.3)
21.5y to 24.0y (ALSPAC); 19.3y to 22.1y (BMDCS)	20.1 (− 7.0 to 47.2)	19.5 (11.1 to 27.8)	–
24.0y to 26.5y (ALSPAC)	15.2 (3.0 to 27.5)	–	–
*Males*			
8.8y to 12.3y (ALSPAC); 5.0y to 8.6y (BMDCS); 7.8y to 12.5y (PBMAS)	148.5 (144.2 to 150.8)	70.2 (61.7 to 78.7)	157.5 (144.2 to 170.8)
12.3y to 15.9y (ALSPAC); 8.6y to 12.3y (BMDCS); 12.5y to 17.1y (PBMAS)	305.6 (302.5 to 308.8)	107.4 (101.7 to 113.1)	247.1 (238.7 to 255.4)
15.9y to 19.4y (ALSPAC); 12.3y to 16.0y (BMDCS); 17.1y to 21.7y (PBMAS)	103.3 (98.6 to 108.0)	253.2 (248.4 to 258.0)	19.3 (8.9 to 29.7)
19.4y to 23.0y (ALSPAC); 16.0y to 19.6y (BMDCS); 21.7y to 26.3y (PBMAS)	93.5 (84.1 to 102.8)	71.4 (63.3 to 79.5)	11.2 (0.8 to 21.6)
23.0y to 26.5y (ALSPAC); 19.6y to 23.3y (BMDCS); 26.3y to 31.0y (PBMAS)	11.7 (−6.4 to 29.9)	8.0 (−3.5 to 19.6)	10.7 (−1.5 to 22.8)
31.0y to 35.6y (PBMAS)	–	–	−7.9 (−26.4 to 10.7)
35.6y to 40.2y (PBMAS)	–	–	−0.4 (−32.7 to 32.0)

Age windows are defined by the number and position of the knots, which were placed at quantiles of the age distribution

**Table 4:** Estimated mean age at peak BMC velocity from the selected natural cubic spline LME models, and selected SITAR models. For comparison, the age windows for peak BMC velocity from the linear spline LME models are also presented

	Females			Males		
	ALSPAC	BMDCS	PBMAS	ALSPAC	BMDCS	PBMAS
*Mean age at peak BMC velocity (years)*						
Natural cubic spline LME model	12.5	12.4	12.2	13.9	14.4	14.1
SITAR model	12.1	12.5	12.0	13.8	14.1	13.9
*age window for peak BMC velocity from the linear spline LME model (years)*	11.4 to 13.9	10.7 to 13.6	7.9 to 14.2	12.3 to 15.9	12.3 to 16.0	12.5 to 17.1

**Fig. 7:**
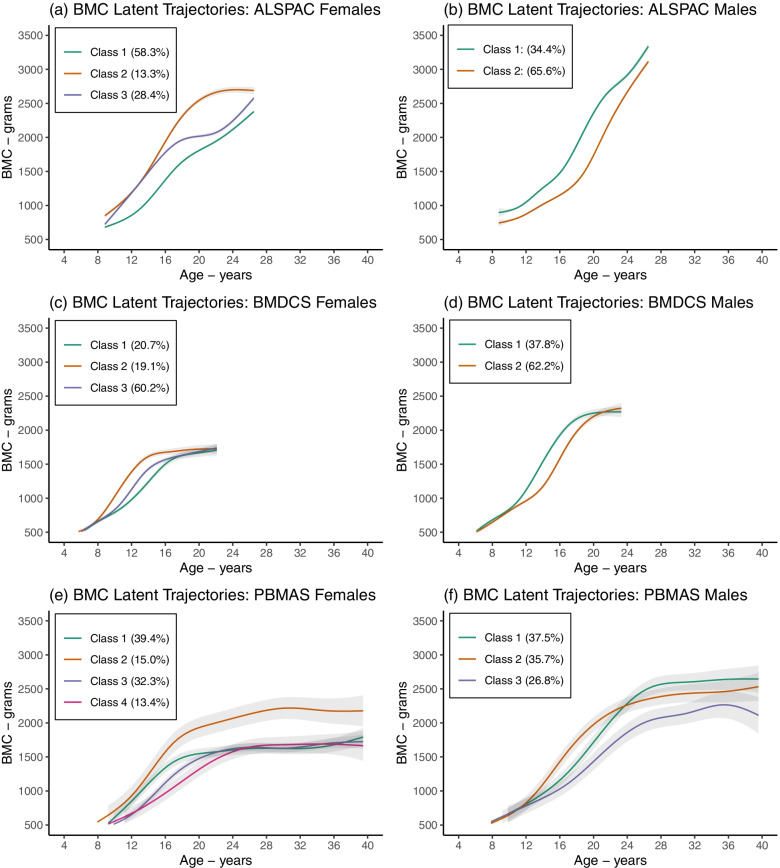
Mean BMC growth trajectories by subgroup (latent class) from the selected growth mixture models. Figure shows the mean BMC growth trajectories by subgroup (latent class) from the selected growth mixture models in ALSPAC females (**a**) and males (**b**), BMDCS females (**c**) and males (**d**), and PBMAS females (**e**) and males (**f**). Colours distinguish between latent trajectory subgroups within subplots and should not be used to compare between subplots. Shaded areas around the mean trajectories represent 95% confidence intervals. The numbers in each class are: ALSPAC females (class 1: *n* = 2337, class 2: *n* = 531, class 3: *n* = 1139), ALSPAC males (class 1: *n* = 1339, class 2: *n* = 2549), BMDCS females (class 1: *n* = 101, class 2: *n* = 93, class 3: *n* = 294), BMDCS males (class 1: *n* = 176, class 2: *n* = 289), PBMAS females (class 1: *n* = 50, class 2: *n* = 19, class 3: *n* = 41, class 4: *n* = 17), PBMAS males (class 1: *n* = 42, class 2: *n* = 40, class 3: *n* = 30)

The selected growth mixture models identified 3 subgroups in females and 2 subgroups in males from ALSPAC and BMDCS, and 4 in females and 3 in males from PBMAS (Fig. 7**)**. Overall, differences in mean BMC between subgroups were larger during adolescence than in childhood and adulthood. One group with 15% of PBMAS females had higher mean BMC up to age 40 than the remaining three groups. A group comprising 27% of PBMAS males reached a lower peak and showed signs of bone loss by age 40, compared to the other two groups. The 2 trajectory groups in ALSPAC males displayed a biologically implausible lack of plateau by early adulthood. Model discrimination capacity was better in PBMAS (and BMDCS) than in ALSPAC, likewise entropy was high in PBMAS and low in ALSPAC and BMDCS (Additional file 3).

## Discussion

Our results provide evidence on bone mineral accrual from 5 to 40 years. Linear and natural cubic spline LME and SITAR showed that the levels and rates of change in BMC were greater for males than females, with peak gains in adolescence in both, but later in males than females. Growth mixture (latent trajectory) models (with trajectory shapes estimated using natural cubic splines) identified potentially distinct trajectory sub-groups, with the greatest between-group differences seen in adolescence. While the main aim of these analyses was to illustrate different modelling approaches, our results are consistent with previous studies on sex differences in BMC and suggest that in both sexes puberty is an important period for peak bone accrual [38–40].

### Choosing a modelling approach

All the modelling techniques presented may be used to model BMC and other growth processes. Both spline LME models and SITAR were previously used on height/weight/BMI [4, 26, 41, 42]; linear splines LME and SITAR models were previously used on bone density [38, 43]; linear and natural cubic spline LME were used to analyse blood pressure change [44, 45]. Choice of method will be determined by research question, including whether this is concerned with differences in mean change over time in the whole study population (where LME or SITAR may be useful), or if the aim is to identify data-driven population subgroups for patterns of change (where a latent trajectory model may be useful). Complexity of the underlying trajectory, and data availability i.e., number of individuals and repeat measurements will also influence choice of method (see [46, 47] for a discussion on sample size in growth models). All models presented can handle unbalanced datasets (i.e., with individually varying measurement occasions, as in our example). If the dataset is balanced (i.e., all measurements taken at the same schedule for everyone, e.g., participants are measured at exactly 2, 4, and 6 years of age), this will limit the complexity of models that can be fitted.

When the main aim is to quantify growth rate at different periods of the life-course, then linear splines may be preferred because of their more interpretable slope coefficients compared to the natural cubic spline LME and SITAR models. If the aim is to describe the shape of the trajectory or identify specific peaks and troughs (e.g., age at peak velocity), then natural cubic spline LME or SITAR are more useful, because linear spline cannot identify points of maxima/minima (but can identify periods/age windows). The number and spacing of repeated measures can influence model convergence and the complexity that can be allowed [48, 49]. SITAR was designed to model adolescent growth in height, and (like other nonlinear models) its parameters reflect its specific purpose. Hence, SITAR may not work well for complex trajectories (e.g., depressive symptoms [50]), and natural cubic spline LME may offer more flexible alternatives. Notable, SITAR fitted without the timing fixed effect (*β*_0_) is analogous to a random intercept random slope model, and so should be at least as flexible as LME. A simple tactic that may improve SITAR (and optimise knot placement in LME models) is transforming the age scale [48, 51].

**Fig. 8:**
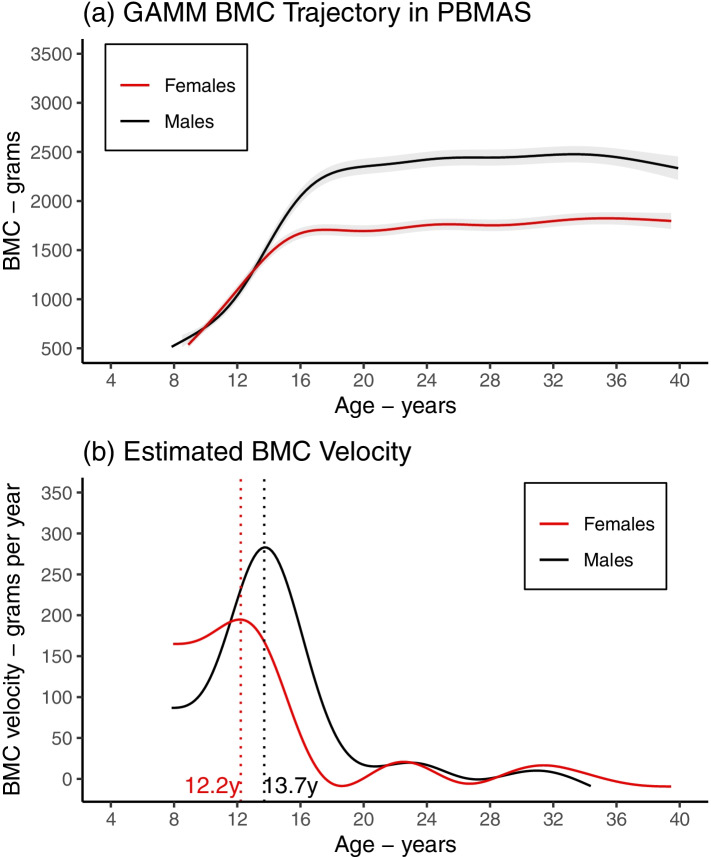
Using a penalised regression spline to describe mean BMC growth trajectory (top panel) and mean BMC growth velocity (bottom panel) in the PBMAS cohort. Figure shows mean BMC growth trajectory (**a**) and mean BMC velocity (**b**) obtained from a penalised regression spline generalised additive mixed model (GAMM) in PBMAS females and males. BMC growth trajectory was parameterised using a low rank (eigen-decomposed) thin plate regression spline. Spline complexity was set at k = 7 and the tuning parameter (λ) was estimated by generalised cross-validation. Growth velocity was estimated by differentiating the mean spline curve. Models included random intercepts and natural cubic spline random slopes. See the documentation for the ‘*mgcv*’ and ‘*gamm4*’ packages for more details on fitting GAMMs and available functionality

The regression splines presented have the advantage of being straightforward to estimate once the number/location of knots is set, with the functions calculated by solving linear equations. If the number of knots is too small, the spline can be sensitive to knot location (and placing knots at quantiles or evenly spaced may not be useful) whereas if the number of knots is too large this can lead to overfitting. Penalised regression splines (introduced in the Methods) are highly flexible alternative functions which avoid the need for knot selection, though are less straightforward to estimate due to the uncertainty of the tuning parameter [12, 16, 17]. One powerful approach for applying penalised regression splines to describe population-average growth trajectories is using generalised additive mixed models (GAMM) [52–55]. Here, the linear predictor depends linearly on an unknown regression spline function of one or more covariates (e.g., time/age) and a tuning parameter is estimated (by REML, generalised cross-validation or other approaches) and used to penalise the spline and avoid overfitting. When applied to PBMAS, a penalised regression spline GAMM estimated mean BMC growth trajectory and velocity curves that were broadly consistent with the curves obtained from regression spline LME and SITAR models (Fig. 8).

Distinct from the LME and SITAR models, we used growth mixture models with a natural cubic spline to identify population subgroups with different nonlinear BMC trajectories. Linear splines with estimated knots [56, 57] and smoothing splines [58] have also been used to parameterise the nonlinear curve in these models (i.e., instead of the natural cubic spline). Latent trajectory models (e.g., growth mixture models) have previously been used to identify trajectory subgroups for BMI [59], depressive symptoms [60], physical activity [61], glucose response [62], and environmental exposures [63], among others. It can be challenging to determine the optimal number of latent classes/sub-groups, including whether the classes are meaningful or if the model is just splitting the distribution of the random effects into a larger group and smaller extremes. Model selection is often subjective, and trajectory subgroups can be cohort-specific and may not replicate in other cohorts therefore, it is important to follow established reporting guidelines [28].

Latent trajectory models will be most useful if there are strong reasons to hypothesise that a study population is composed of multiple unknown sub-groups of individuals with distinct trajectories. Less complex latent trajectory models (e.g., group-based trajectory models) are computationally more efficient than growth mixture models though, any subgroup differences identified by such models may just reflect within class variability, which is likely to be absorbed by random effects in growth mixture models. If the aim was in exploring specific hypotheses (e.g., sensitive periods), then clustering (data driven) approaches may not provide a suitable sub-group to test this, e.g., if the aim was to explore the hypothesis that a lower birth weight followed by faster growth in the first 1000 days increased cardiovascular risk, this approach might not identify a cluster with this specific growth pattern. There may also be value in using a combination of approaches [64], like in this paper.

### Identifying determinants and outcomes of trajectories

**Fig. 9:**
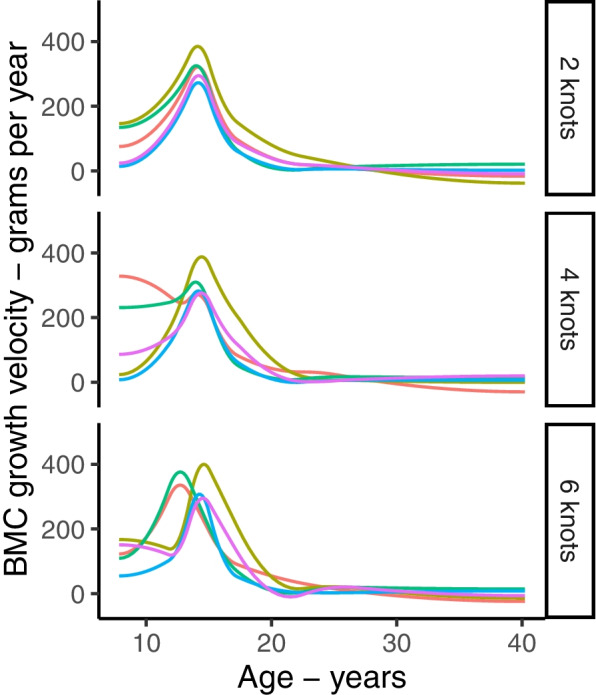
Effect of increasing the number of knots in the random effects spline on the individual growth velocity curves from the natural cubic spline LME model. Figure shows velocity curves for 5 randomly selected individuals, obtained from natural cubic spline LME models in PBMAS males with 2, 4, and 6 knots for the random effects spline curve. All models included 6 knots in the fixed effects spline curve. All knots were placed at quantiles of age distribution

The models in this paper can be extended to include early life exposures and later outcomes to explore their associations with trajectories. Choice of method will depend on the research aims. Exposure variables can be added within LME spline models as fixed effects and as interactions with splines to test their effect on trajectories and growth rate [3, 38, 65]. The individual growth features (e.g., peak velocity and age at peak velocity) can be obtained from spline LME model random effects and used in separate analyses as outcomes or exposures [18, 66] – however**,** it is important to allow enough complexity in the random-effects splines for sufficient between-person variability (Fig. 9). Individual growth features can be easily obtained from SITAR and used in subsequent analyses to examine associations with exposures or outcomes [43, 67]. Of note, 2-stage approaches may be more biased than 1-stage joint model [68, 69]. Exposures and outcomes can be related to latent trajectories in a joint model or a multistage process where subgroups are first identified and subsequently used in separate models (unweighted or (preferably) weighed for classification probabilities) to examine associations [30, 59–63]. If the aim was to identify effects of repeated exposure, then a ‘structured’ modelling approach may be useful for testing competing hypotheses [70].

It is important to identify potential biases and explore ways for mitigating them when analysing (causal) associations between trajectories and exposures or outcomes in cohort studies. Missing data can bias associations depending on mechanism, and appropriate approaches to describe and handle missing data should be explored [12, 71–76]. In a repeated measure setting, individuals with missing outcome values can be included in the estimation sample if they have at least one observed outcome value. Mixed-effects models give unbiased results (i.e., not biased by missing data) when the probability that an outcome value is missing depends on observed values of the outcome (i.e., outcome missing at random (MAR) depending on observed outcome values). Bias due to missing data will occur for these models when the probability that the outcome is missing depends on underlying missing values (i.e., outcome missing not at random (MNAR) depending on missing outcome values). With incomplete covariates, bias can occur when the probability of excluding an individual with missing covariate data is related to the outcome. Regardless of bias, excluding individuals with missing covariate information will often mean discarding useful observed data, leading to imprecision [77].

Confounding can lead to spurious associations between trajectories and exposures or outcomes. Confounders (factors causally related to exposure and outcome) should be identified (e.g., using Directed Acyclic Graphs) and controlled for, taking care not to adjust for mediators (factors on the causal pathways) [78, 79]. Even with adjustment, residual confounding (from using poorly measured confounders or not adjusting for important confounders) can bias results. Some useful strategies for checking if residual confounding influences results include using negative control variables and comparing results across cohorts with different confounding structures [64, 80–82].

### Comparing and modelling trajectories across cohorts

**Fig. 10:**
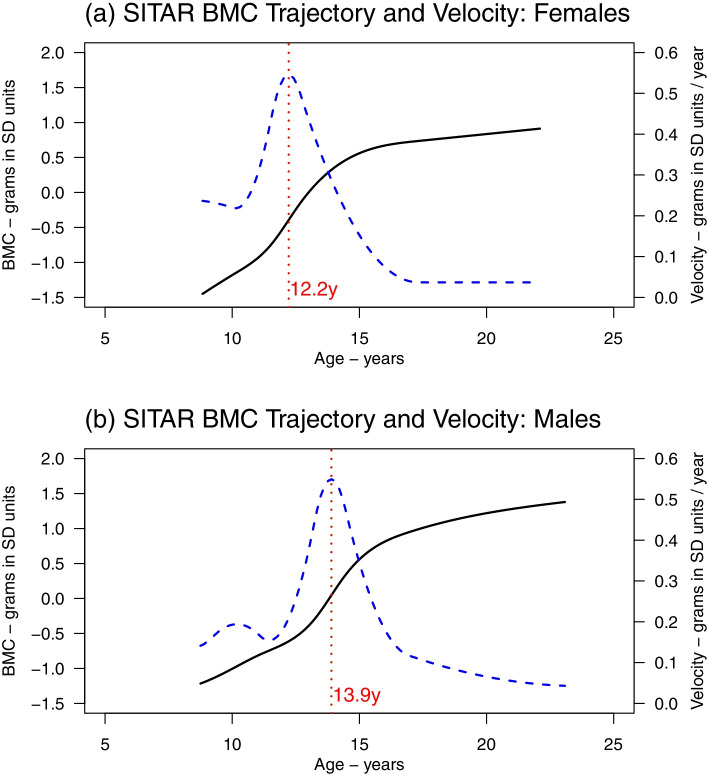
Pooled mean BMC growth trajectory (solid black curves), mean BMC growth velocity (dashed blue curves), and mean age at peak BMC velocity (vertical red lines) from SITAR models applied to individual participant data (ALSPAC, BMDCS and PBMAS). Figure shows mean BMC growth trajectory (solid black curves), mean BMC growth velocity (dashed blue curves), and mean age at peak BMC velocity (vertical red lines) from SITAR models applied to individual participant data (ALSPAC, BMDCS and PBMAS) for females (a) and males (b). Sex-specific individual participant data SITAR models were fitted to ALSPAC, BMDCS and PBMAS combined, to obtained pooled estimates of the timing of peak BMC growth. This analysis included individuals with overlapping measurements (8.8 to 22.1 years) from the 3 cohorts (n=4431 for females and n=4359 for males.) To mitigate the cohort differences in BMC (higher values in ALSPAC due to Lunar machine), we modelled BMC in cohort-specific standardised units (mean=0 and SD=1), and the models were adjusted for cohort (as a fixed effect). Note it is not advised to fit SITAR to SD units as this distorts the underlying biology – though in our example, results are consistent with cohort-specific natural unit results

Researchers should be aware of potential differences in the participants, data collection methods and analysis models when comparing trajectories from different studies. For example, the higher BMC due to the Lunar machine in ALSPAC means it is inaccurate to conclude that Britons had higher BMC (and peak BMC velocity) than North Americans. Another example is the effect of medication use by older cohorts on combined blood pressure trajectories [83]. New and existing multicohort collaborations provide unique opportunities to jointly model such trajectories across different cohorts and to extend the amount of the life course studied [84, 85]. However, this also generates additional challenges including on model selection and missing data (see [86] for a discussion of challenges and solutions to multicohort modelling). Whatever approach is taken (cohort-specific or multicohort modelling), data harmonisation is an important initial step that involves making data comparable across studies [87, 88], e.g. using DXA reference standards to harmonise BMC [89]. Because age (but not BMC) was fully harmonised in our example, a simple approach to obtaining valid pooled estimate of age at peak BMC velocity is to fit a growth model to all individuals with BMC expressed in cohort-specific SD units (Fig. 10).

## Conclusions

LME models with linear and natural cubic splines, SITAR, and growth mixture models are useful for describing nonlinear growth trajectories in longitudinal population studies, and these methods can be adapted to other complex traits. Choice of method depends on research aims, complexity of the trajectory, and available data. This illustrative paper and accompanying R analysis code and example datasets will we hope be a useful resource for researchers interested in modelling nonlinear longitudinal trajectories.

## Supplementary Information

**Additional file 1.** Cohort characteristics and participant numbers, and age and BMC at each visit.

**Additional file 2.** BIC and fit statistics for linear spline LME models, natural cubic spline LME models, and SITAR models with 2 to 6 knots in the fixed effects spline curve.

**Additional file 3.** Fit statistics and predicted mean BMC latent trajectories for growth mixture models with 1 to 5 latent classes.

## Data Availability

All the analysis code used in this paper can be found at https://github.com/aelhak/nltmr/. Details of all available data and the processes and procedures involved in accessing the ALSPAC resource can be found in the ALSPAC study website, which includes a fully searchable data dictionary and variable search tool (http://www.bristol.ac.uk/alspac/researchers/our-data/). The BMDCS data can be accessed through the NICHD DASH website (https://dash.nichd.nih.gov/). Researchers interested in accessing the PBMAS data should contact Professor Adam DG Baxter-Jones (baxter.jones@usask.ca).

## Abbreviations

ALSPAC: Avon Longitudinal Study of Parents and Children
BMC: Bone mineral content
BMDCS: Bone Mineral Density in Childhood Study
BMI: Body mass index
DXA: Dual-Energy X-ray Absorptiometry
GAMM: Generalised additive mixed model
GEE: Generalised estimating equations
LME: Linear mixed effects
MAR: Missing at random
ML: Maximum likelihood
MNAR: Missing not at random
PBMAS: Pediatric Bone Mineral Accrual Study
REML: Restricted maximum likelihood
SITAR: Super Imposition by Translation and Rotation
