# Supplementary material for: Using linear and natural cubic splines, SITAR, and latent trajectory models to characterise nonlinear longitudinal growth trajectories in cohort studies
Source: BMC Med Res Methodol. 2022 Mar 15;22:68. doi: 10.1186/s12874-022-01542-8 (PMC8925070; doi:10.1186/s12874-022-01542-8)
Supplement: Supplementary file 1 — Additional file 1. Cohort characteristics and participant numbers, and age and BMC at each visit. [file 12874_2022_1542_MOESM1_ESM.docx]

# **Additional file 1** Cohort characteristics and participant numbers, age, and BMC at each visit

**Additional file 1a** Cohort characteristics and supporting information

ALSPAC is a large birth cohort study that recruited all pregnant women residing in southwest England with an expected date of delivery between 1991-1992. In total, 15 247 eligible pregnancies were enrolled in ALSPAC (75% response), resulting in 14 973 live births, of whom 14 899 were alive at 1 year of age. 98% of ALSPAC participants were of European descent. Detailed information was collected from offspring and parents using questionnaires, data extraction from medical records, linkage to health records, and dedicated clinic assessments. Details of all available data can be found in the ALSPAC study website (<http://www.bristol.ac.uk/alspac/>), which includes a fully searchable data dictionary and variable search tool. All ALSPAC offspring were invited to undergo whole-body dual-energy x-ray absorptiometry (DXA) scans as part of clinic assessments at approximate ages 10, 12, 14, 16, 18, and 25 years. DXA scans were performed using a Lunar Prodigy scanner (Lunar Radiation Corp) and analysed according to manufacturer’s standard scanning software and positioning protocols. Scans were reanalysed as necessary to ensure optimal placement of borders between adjacent subregions. Total-body (minus head) BMC (measured in grams) was extracted from each scan. ALSPAC data were collected and managed using REDCap electronic data capture tools hosted at the University of Bristol (PA Harris, R Taylor, R Thielke, J Payne, N Gonzalez, JG. Conde, Research electronic data capture (REDCap) – A metadata-driven methodology and workflow process for providing translational research informatics support. J Biomed Inform. 2009 Apr;42(2):377-81). REDCap (Research Electronic Data Capture) is a secure, web-based software platform designed to support data capture for research studies. Ethics approval was obtained from the ALSPAC law and ethics committee and the local National Health Service research ethics committee. Written informed consent was obtained from all participants.

BMDCS is a multi-ethnic cohort of children aged 5-19 years at study entry enrolled at 5 US centres. Older (age 19 years) and younger (age 5 years) participants were recruited in 2006–2007 and were followed annually for 2 years (maximum 3 visits). All BMDCS participants underwent up to 7 annual whole-body DXA scans (Hologic QDR4500A, Bedford, MA, USA) between 2002-2010. Scans were performed according to manufacturer’s guidelines by trained research technicians. Scans were analysed by the DXA Core Laboratory (University of San Francisco, San Francisco, CA, USA) using Hologic software (v.12.3) for baseline scans and Apex 2.1 for follow-up scans. Scans were adjusted for calibration differences among clinical centers and longitudinal drift. Total-body (minus head) BMC (measured in grams) was extracted from each scan. Individuals 18 years and older gave written informed consent. Parental or guardian consent plus participant assent were obtained for individuals younger than 18 years old. The study was approved by the Institutional Review Board of each respective clinical center.

PBMAS is a long running cohort study initiated in 1991 which recruited children aged 8-15 years and residing in Saskatchewan, Canada. 220 participants (107 boys and 113 girls) were recruited from schools in the city of Saskatoon (population 200,000). Between 1992 and 1993, a further 5 boys and 18 girls, aged 8 to 10 years, were recruited. Eligible children had no history of chronic disease or long‐term medication use. 95% of PBMAS participants were of European descent. PBMAS carried out up to 16 DXA scans (Hologic QDR-2000, array mode) between 1991 and 2017. After an initial 7 yearly scans between 1991-1998, scans were repeated between 2003-2005, 2007-2011 and 2016-2017. Scans were done by a trained technician following the procedures outlined in the operator's manual and user guide. Scans were analysed using software version 5.67A. Total-body (minus head) BMC (measured in grams) was extracted from each scan. Written consent was obtained from all participants. All procedures were approved by the University of Saskatchewan's biomedical review committee.

To be included in the trajectory modelling, individuals from each study had to have at least one measure of BMC and no missing data on age or sex. To simplify the illustrative examples and because ALSPAC and PBMAS were both ethnically homogenous, we restricted our analysis to white individuals. Likely outlying observations were removed if BMC was 600 grams or more lower than in the previous observation and was measured before age 30 (n=4 observations were removed (2 in ALSPAC females; 1 in ALSPAC males; 1 in PBMAS males). Our final analysis sample included 3,888 males and 4,007 females in ALSPAC, 465 males and 488 females in BMDCS, and 112 males and 127 females in PBMAS with ≥1 BMC measurement. Median [interquartile range] numbers of repeated BMC measurements per individual were 4 [3], 6 [4] and 10 [7] in ALSPAC, BMDCS and PBMAS, respectively. Online Resource 1b presents the numbers of participants with BMC data at each visit and the mean, standard deviation, minimum and maximum age and BMC

**Additional file 1b** Numbers of participants with BMC data at each visit and mean, standard deviation, minimum and maximum age and BMC

| **cohort** | **sex** | **visit** | **N** | **age_mean** | **age_sd** | **age_min** | **age_max** | **tblh_bmc_mean** | **tblh_bmc_sd** | **tblh_bmc_min** | **tblh_bmc_max** |
| --- | --- | --- | --- | --- | --- | --- | --- | --- | --- | --- | --- |
| ALSPAC | Females | 1 | 3301 | 9.9 | 0.3 | 8.8 | 11.7 | 878.6 | 189 | 436.4 | 1933.1 |
| ALSPAC | Females | 2 | 3170 | 11.7 | 0.2 | 10.4 | 13.6 | 1235.7 | 293.8 | 605.8 | 2422.8 |
| ALSPAC | Females | 3 | 2751 | 13.8 | 0.2 | 12.6 | 15.2 | 1720.7 | 341.3 | 790.6 | 3439.3 |
| ALSPAC | Females | 4 | 2254 | 15.5 | 0.4 | 14.2 | 17.5 | 1920.6 | 343.4 | 1034.1 | 3541.3 |
| ALSPAC | Females | 5 | 2395 | 17.8 | 0.4 | 16.2 | 20 | 2044.2 | 369.8 | 1100.2 | 3578.3 |
| ALSPAC | Females | 6 | 1980 | 24.4 | 0.8 | 22.4 | 26.5 | 2340.1 | 273.3 | 1591.1 | 3454.2 |
| ALSPAC | Males | 1 | 3246 | 9.9 | 0.3 | 8.8 | 11.7 | 903.7 | 173.5 | 409.4 | 1777.8 |
| ALSPAC | Males | 2 | 3102 | 11.7 | 0.2 | 10.8 | 13.5 | 1184.5 | 249.5 | 500.1 | 2384.1 |
| ALSPAC | Males | 3 | 2657 | 13.8 | 0.2 | 12.5 | 15.1 | 1720.5 | 405.4 | 639.9 | 3564 |
| ALSPAC | Males | 4 | 1986 | 15.4 | 0.3 | 14.5 | 17.7 | 2204.6 | 447.9 | 806.5 | 3875.7 |
| ALSPAC | Males | 5 | 1900 | 17.8 | 0.4 | 16.4 | 19.9 | 2565.2 | 464.2 | 1157.8 | 4235.1 |
| ALSPAC | Males | 6 | 1201 | 24.5 | 0.8 | 22.4 | 26.5 | 3048.8 | 403.2 | 1954 | 4610.9 |
| BMDCS | Females | 1 | 367 | 10.7 | 2.9 | 6 | 16 | 962.4 | 404.7 | 403.2 | 1954.6 |
| BMDCS | Females | 2 | 356 | 11.7 | 3 | 6.9 | 17.1 | 1070.2 | 417.8 | 437.7 | 2008.3 |
| BMDCS | Females | 3 | 334 | 12.7 | 2.9 | 7.9 | 18 | 1171.6 | 410 | 485 | 2127 |
| BMDCS | Females | 4 | 329 | 13.7 | 2.9 | 8.9 | 19 | 1280.1 | 404 | 552.7 | 2281.8 |
| BMDCS | Females | 5 | 412 | 13.9 | 4.6 | 5 | 20 | 1273.6 | 483.8 | 346.2 | 2228.7 |
| BMDCS | Females | 6 | 415 | 15 | 4.6 | 5.9 | 21.4 | 1346.2 | 460.8 | 399.4 | 2277.2 |
| BMDCS | Females | 7 | 412 | 16 | 4.6 | 6.9 | 22.3 | 1417.6 | 433.9 | 455.8 | 2352.2 |
| BMDCS | Males | 1 | 353 | 10.9 | 3.2 | 6 | 17 | 1052 | 542.8 | 362.3 | 2843.6 |
| BMDCS | Males | 2 | 343 | 12 | 3.2 | 7 | 18 | 1195.9 | 581.3 | 415.4 | 2980.4 |
| BMDCS | Males | 3 | 329 | 13 | 3.2 | 8 | 19 | 1328.7 | 596 | 503.6 | 3237.2 |
| BMDCS | Males | 4 | 312 | 14 | 3.2 | 8.9 | 19.9 | 1474.7 | 621.9 | 574.8 | 3504.2 |
| BMDCS | Males | 5 | 400 | 14.2 | 4.7 | 5 | 21.3 | 1523.4 | 696.7 | 343.4 | 3525 |
| BMDCS | Males | 6 | 380 | 15.1 | 4.8 | 5.9 | 22.4 | 1607.2 | 693.5 | 404.2 | 3478.7 |
| BMDCS | Males | 7 | 366 | 16.1 | 4.7 | 6.9 | 23.3 | 1724.8 | 663.2 | 468.8 | 3441.2 |
| PBMAS | Females | 1 | 110 | 11.7 | 1.9 | 8 | 14.9 | 1050.6 | 431.2 | 330.8 | 2192.4 |
| PBMAS | Females | 2 | 117 | 12.2 | 2.3 | 7.9 | 16 | 1107.3 | 459.4 | 361.5 | 2197.7 |
| PBMAS | Females | 3 | 115 | 13.1 | 2.4 | 8.1 | 16.9 | 1237.4 | 456.3 | 433.2 | 2251.3 |
| PBMAS | Females | 4 | 100 | 13.8 | 2.3 | 9.1 | 17.9 | 1333.9 | 423.8 | 527.4 | 2383.8 |
| PBMAS | Females | 5 | 97 | 14.7 | 2.3 | 10.1 | 18.9 | 1450.5 | 391.8 | 626.2 | 2474.2 |
| PBMAS | Females | 6 | 84 | 15.5 | 2.3 | 11.1 | 19.9 | 1526.1 | 374 | 733.9 | 2581.7 |
| PBMAS | Females | 7 | 61 | 16.3 | 2.3 | 12.1 | 21 | 1596.5 | 331.6 | 998.5 | 2634 |
| PBMAS | Females | 8 | 7 | 14.5 | 0.7 | 13.5 | 15.1 | 1475.6 | 149.2 | 1273.4 | 1648.2 |
| PBMAS | Females | 9 | 78 | 21.9 | 2.3 | 17.3 | 26.1 | 1742.6 | 326.5 | 1179.4 | 3073.9 |
| PBMAS | Females | 10 | 74 | 23.1 | 2.3 | 18.3 | 27.2 | 1749.2 | 331.7 | 1203.2 | 2912.9 |
| PBMAS | Females | 11 | 70 | 23.9 | 2.3 | 19.3 | 28.5 | 1722.6 | 298.1 | 1203.4 | 2496.2 |
| PBMAS | Females | 12 | 73 | 25.6 | 2.3 | 21.1 | 30.1 | 1749.9 | 343.3 | 1162.3 | 2908.7 |
| PBMAS | Females | 13 | 59 | 29.3 | 2.4 | 24.4 | 33.2 | 1789.7 | 354.6 | 1213 | 2826.5 |
| PBMAS | Females | 14 | 39 | 30.7 | 2.3 | 26.2 | 34.6 | 1826.6 | 306.1 | 1262.2 | 2580.9 |
| PBMAS | Females | 15 | 19 | 36.1 | 2.7 | 31.2 | 39.5 | 1897.2 | 388.6 | 1464.4 | 2872.7 |
| PBMAS | Females | 16 | 10 | 35.8 | 2.1 | 31.5 | 38.9 | 1723.6 | 271.9 | 1287.6 | 2157.3 |
| PBMAS | Males | 1 | 107 | 11.8 | 1.8 | 8 | 15.1 | 1091.9 | 502.3 | 370.5 | 2829.5 |
| PBMAS | Males | 2 | 105 | 12.5 | 2 | 7.8 | 16.1 | 1195 | 505.6 | 367.8 | 2560.2 |
| PBMAS | Males | 3 | 103 | 13.5 | 2 | 8.8 | 17.2 | 1420.1 | 552 | 462.8 | 2769 |
| PBMAS | Males | 4 | 86 | 14.3 | 2.1 | 9.8 | 18.1 | 1595.7 | 588.6 | 557.6 | 2861.1 |
| PBMAS | Males | 5 | 83 | 15.3 | 2.1 | 10.8 | 19.1 | 1794.8 | 559.2 | 649.6 | 2910.7 |
| PBMAS | Males | 6 | 69 | 16.2 | 2.1 | 11.8 | 20.1 | 2000.6 | 535.7 | 718.5 | 3007.9 |
| PBMAS | Males | 7 | 51 | 16.9 | 2.2 | 12.8 | 20.7 | 2104.5 | 525.7 | 866.1 | 3064 |
| PBMAS | Males | 8 | 5 | 15.5 | 1 | 14.5 | 17 | 1722.8 | 499.8 | 1089.1 | 2220.4 |
| PBMAS | Males | 9 | 60 | 22.6 | 2.2 | 18.1 | 26.8 | 2416.2 | 376.6 | 1587.7 | 3172 |
| PBMAS | Males | 10 | 56 | 23.7 | 2.2 | 19.2 | 27.4 | 2440.1 | 368.2 | 1540.4 | 3198.2 |
| PBMAS | Males | 11 | 56 | 24.4 | 2.3 | 19.9 | 28.1 | 2444.2 | 358.3 | 1705.8 | 3239.6 |
| PBMAS | Males | 12 | 56 | 26.4 | 2.2 | 21.9 | 30 | 2457 | 360 | 1705.8 | 3157.6 |
| PBMAS | Males | 13 | 34 | 29.9 | 2.3 | 25.3 | 33.7 | 2516.1 | 371.4 | 1897.4 | 3222.4 |
| PBMAS | Males | 14 | 35 | 31.4 | 2.1 | 26.5 | 35 | 2561.9 | 351.5 | 1828.2 | 3158.4 |
| PBMAS | Males | 15 | 12 | 34.9 | 2 | 32.2 | 38.8 | 2459.1 | 439.7 | 1886.7 | 3120 |
| PBMAS | Males | 16 | 14 | 37.3 | 1.9 | 34.3 | 40.2 | 2503.2 | 223.8 | 2189.4 | 2866.8 |
