# Supplementary material for: Using linear and natural cubic splines, SITAR, and latent trajectory models to characterise nonlinear longitudinal growth trajectories in cohort studies
Source: BMC Med Res Methodol. 2022 Mar 15;22:68. doi: 10.1186/s12874-022-01542-8 (PMC8925070; doi:10.1186/s12874-022-01542-8)
Supplement: Supplementary file 2 — Additional file 2. BIC and fit statistics for linear spline LME models, natural cubic spline LME models, and SITAR models with 2 to 6 knots in the fixed effects spline curve. [file 12874_2022_1542_MOESM2_ESM.docx]

# **Additional file 2** BIC and fit statistics for linear spline LME models, natural cubic spline LME models, and SITAR models with 2 to 6 knots in the fixed effects spline curve

**Additional file 2a** BIC values for linear spline LME models: data show the model with the smallest BIC value and difference from the smallest BIC for each model

|  | Number of knots in mean spline curve | ALSPAC | BMDCS | PBMAS |
| --- | --- | --- | --- | --- |
| Females |  |  |  |  |
|  | 2 | 388.3 | 414.2 | 760.5 |
|  | 3 | 2,659.6 | 609.0 | 150.2 |
|  | 4 | 1187.8 | 342.4 | min (13692.2) |
|  | 5 | 120.7 | min (30131.6) | 140.0 |
|  | 6 | min (208519.3) | 43.9 | 205.1 |
| Males |  |  |  |  |
|  | 2 | 3473.4 | 302.4 | 232.4 |
|  | 3 | 3082.2 | 1258.1 | 186.0 |
|  | 4 | min (188966.9) | min (29851.0) | 356.3 |
|  | 5 | 841.6 | 410.7 | 241.51 |
|  | 6 | 284.5 | 115.0 | min (12041.00) |

**Additional file 2b** BIC values for natural cubic spline LME models: data show the model with the smallest BIC value and difference from the smallest BIC for each model

|  | Number of knots in mean spline curve | ALSPAC | BMDCS | PBMAS |
| --- | --- | --- | --- | --- |
| Females |  |  |  |  |
|  | 2 | 2582.7 | 607.7 | 2670.5 |
|  | 3 | min (208027.1) | 231.7 | 242.7 |
|  | 4 | 61.6 | 94.4 | 80.5 |
|  | 5 | 11.4 | min (30240.9) | 6.4 |
|  | 6 | 1.4 | 9.3 | min (13550.3) |
| Males |  |  |  |  |
|  | 2 | 932.6 | 516.5 | 625.5 |
|  | 3 | 795.7 | 381.3 | 129.9 |
|  | 4 | 580.6 | 92.9 | 13.0 |
|  | 5 | 10.8 | min (29959.7) | 15.1 |
|  | 6 | min (188048.7) | 6.7 | min (11860.2) |

**Additional file 2c** BIC values for SITAR models: data show the model with the smallest BIC value and difference from the smallest BIC for each model

|  | Number of knots in mean spline curve | ALSPAC | BMDCS | PBMAS |
| --- | --- | --- | --- | --- |
| Females |  |  |  |  |
|  | 2 | min (207809.5) | 82.3 | min (13329.3) |
|  | 3 | - | 21.3 | 12.3 |
|  | 4 | - | 29.0 | 19.1 |
|  | 5 | - | min (29017.1) | 5.8 |
|  | 6 | - | 2.9 | 77.0 |
| Males |  |  |  |  |
|  | 2 | - | 70.6 | 19.9 |
|  | 3 | - | 74.4 | 1.6 |
|  | 4 | 365.5 | min (29023.3) | min (11524.4) |
|  | 5 | min (185730.8) | 5.2 | 0.1 |
|  | 6 | 424.1 | 33.4 | 7.2 |

**Additional file 2d** Observed BMC, predicted BMC (with random effects) and difference between observed and predicted values from the linear spline LME models.

|  | N | Observed BMC | Predicted BMC | Observed – predicted BMC |
| --- | --- | --- | --- | --- |
| ALSPAC females |  |  |  |  |
| Visit 1 | 3301 | 878.6 (189.0) | 879.6 (200.5) | -1.1 (53.9) |
| Visit 2 | 3170 | 1235.7 (293.8) | 1234.7 (243.1) | 1 (96.0) |
| Visit 3 | 2751 | 1720.7 (286.3) | 1722.2 (286.3) | -1.5 (99.0) |
| Visit 4 | 2254 | 1920.6 (319.6) | 1916.8 (319.6) | 3.9 (76.0) |
| Visit 5 | 2395 | 2044.2 (370.0) | 2045.9 (370.0) | -1.8 (86.0) |
| Visit 6 | 1980 | 2340.1 (243.0) | 2340.2 (243.0) | 0 (73.6) |
| ALSPAC males |  |  |  |  |
| Visit 1 | 3246 | 903.7 (173.5) | 905.1 (154.9) | -1.3 (68.5) |
| Visit 2 | 3102 | 1184.5 (249.5) | 1181.6 (237.9) | 2.9 (82.0) |
| Visit 3 | 2657 | 1720.5 (405.4) | 1724.3 (330.8) | -3.8 (137.1) |
| Visit 4 | 1986 | 2204.6 (447.9) | 2202.1 (399.2) | 2.6 (105.2) |
| Visit 5 | 1900 | 2565.2 (464.2) | 2565 (473.3) | 0.1 (128.1) |
| Visit 6 | 1201 | 3048.8 (403.2) | 3048.8 (369.5) | 0 (61.3) |
| BMDCS females |  |  |  |  |
| Visit 1 | 367 | 962.4 (404.7) | 959.3 (400.8) | 3.1 (41.7) |
| Visit 2 | 356 | 1070.2 (417.8) | 1070.7 (414.1) | -0.5 (32.5) |
| Visit 3 | 334 | 1171.6 (410.0) | 1175.1 (407.6) | -3.5 (37.5) |
| Visit 4 | 329 | 1280.1 (404.0) | 1280.1 (400.7) | 0 (40.8) |
| Visit 5 | 412 | 1273.6 (438.8) | 1275.2 (482.2) | -1.6 (32.9) |
| Visit 6 | 415 | 1346.2 (460.8) | 1346.2 (456.0) | 0 (27.1) |
| Visit 7 | 412 | 1417.6 (433.9) | 1415.5 (433.5) | 2.1 (29.8) |
| BMDCS males |  |  |  |  |
| Visit 1 | 353 | 1052 (542.8) | 1053.6 (541.2) | -1.5 (45.6) |
| Visit 2 | 343 | 1195.9 (581.3) | 1200.2 (583.8) | -4.3 (38.2) |
| Visit 3 | 329 | 1328.7 (596.0) | 1321.9 (591.6) | 6.8 (42.6) |
| Visit 4 | 312 | 1474.7 (621.9) | 1465.4 (604.6) | 9.3 (65.2) |
| Visit 5 | 400 | 1523.4 (696.7) | 1531.8 (696.0) | -8.4 (48.6) |
| Visit 6 | 380 | 1607.2 (693.5) | 1607.5 (691.4) | -0.3 (35.1) |
| Visit 7 | 366 | 1724.8 (663.2) | 1723.9 (661.9) | 0.8 (45.4) |
| PBMAS females |  |  |  |  |
| Visit 1 | 110 | 1050.6 (431.2) | 1043.7 (450.9) | 7 (84.9) |
| Visit 2 | 117 | 1107.3 (459.4) | 1116.6 (475.9) | -9.3 (68.8) |
| Visit 3 | 115 | 1237.4 (456.3) | 1241.2 (447.3) | -3.7 (55.3) |
| Visit 4 | 100 | 1333.9 (423.8) | 1339.4 (393.9) | -5.5 (63.7) |
| Visit 5 | 97 | 1450.5 (391.8) | 1445.9 (356.4) | 4.6 (71.4) |
| Visit 6 | 84 | 1526.1 (374.0) | 1519.9 (331.4) | 6.2 (85.1) |
| Visit 7 | 61 | 1596.5 (331.6) | 1581.9 (292.2) | 14.6 (87.6) |
| PBMAS males |  |  |  |  |
| Visit 1 | 107 | 1091.9 (502.3) | 1084.5 (486.6) | 7.3 (95.9) |
| Visit 2 | 105 | 1195 (505.6) | 1196.7 (493.6) | -1.6 (83.4) |
| Visit 3 | 103 | 1420.1 (552.0) | 1411 (528.4) | 9.1 (84.5) |
| Visit 4 | 86 | 1595.7 (588.6) | 1604.1 (571.3) | -8.4 (89.4) |
| Visit 5 | 83 | 1794.8 (559.2) | 1813.9 (550.2) | -19.1 (99.9) |
| Visit 6 | 69 | 2000.6 (535.7) | 2004.2 (524.8) | -3.6 (125.3) |
| Visit 7 | 51 | 2104.5 (525.7) | 2096 (491.5) | 8.5 (149.7) |

Data are means (SD). For brevity, results for the first 7 visits only are presented for PBMAS

**Additional file 2e** Observed BMC, predicted BMC (with random effects) and difference between observed and predicted BMC from the natural cubic spline LME models.

|  | N | Observed BMC | Predicted BMC | Observed – predicted BMC |
| --- | --- | --- | --- | --- |
| ALSPAC females |  |  |  |  |
| Visit 1 | 3301 | 878.6 (189.0) | 879.5 (180.3) | -1 (55.4) |
| Visit 2 | 3170 | 1235.7 (293.8) | 1235.5 (251.1) | 0.2 (99.7) |
| Visit 3 | 2751 | 1720.7 (341.3) | 1718 (307.2) | 2.7 (89.4) |
| Visit 4 | 2254 | 1920.6 (343.4) | 1921.5 (330.1) | -0.8 (78.3) |
| Visit 5 | 2395 | 2044.2 (369.8) | 2047.3 (349.0) | -3.1 (101.5) |
| Visit 6 | 1980 | 2340.1 (273.3) | 2338 (247.1) | 2.1 (67.7) |
| ALSPAC males |  |  |  |  |
| Visit 1 | 3246 | 903.7 (173.5) | 903.9 (135.5) | -0.1 (74.9) |
| Visit 2 | 3102 | 1184.5 (249.5) | 1184.4 (254.0) | 0.1 (82.4) |
| Visit 3 | 2657 | 1720.5 (405.4) | 1720.8 (359.2) | -0.3 (114.3) |
| Visit 4 | 1986 | 2204.6 (447.9) | 2203.1 (409.7) | 1.6 (93.4) |
| Visit 5 | 1900 | 2565.2 (464.2) | 2567.2 (441.7) | -2 (122.3) |
| Visit 6 | 1201 | 3048.8 (403.2) | 3047.6 (386.6) | 1.3 (36.5) |
| BMDCS females |  |  |  |  |
| Visit 1 | 367 | 962.4 (404.7) | 958.7 (401.2) | 3.7 (46.9) |
| Visit 2 | 356 | 1070.2 (417.8) | 1068.6 (413.5) | 1.6 (31.3) |
| Visit 3 | 334 | 1171.6 (410.0) | 1174.5 (407.1) | -2.9 (38.9) |
| Visit 4 | 329 | 1280.1 (404.0) | 1281.6 (399.4) | -1.5 (45.0) |
| Visit 5 | 412 | 1273.6 (483.8) | 1278.8 (481.6) | -5.2 (33.9) |
| Visit 6 | 415 | 1346.2 (460.8) | 1346.7 (456.7) | -0.5 (29.2) |
| Visit 7 | 412 | 1417.6 (433.9) | 1413 (432.5) | 4.6 (33.4) |
| BMDCS males |  |  |  |  |
| Visit 1 | 353 | 1052 (542.8) | 1049.4 (533.7) | 2.6 (58.7) |
| Visit 2 | 343 | 1195.9 (581.3) | 1200.4 (581.6) | -4.4 (42.0) |
| Visit 3 | 329 | 1328.7 (596.0) | 1326.9 (594.7) | 1.9 (45.8) |
| Visit 4 | 312 | 1474.7 (621.9) | 1469.3 (608.3) | 5.4 (71.0) |
| Visit 5 | 400 | 1523.4 (696.7) | 1529.4 (696.8) | -6 (53.4) |
| Visit 6 | 380 | 1607.2 (693.5) | 1605.3 (689.9) | 1.9 (37.2) |
| Visit 7 | 366 | 1724.8 (663.2) | 1724.9 (660.5) | -0.1 (50.7) |
| PBMAS females |  |  |  |  |
| Visit 1 | 110 | 1050.6 (431.2) | 1043.1 (431.8) | 7.5 (70.8) |
| Visit 2 | 117 | 1107.3 (459.4) | 1115.9 (463.7) | -8.6 (50.3) |
| Visit 3 | 115 | 1237.4 (456.3) | 1236.9 (452.1) | 0.5 (49.8) |
| Visit 4 | 100 | 1333.9 (423.8) | 1338.9 (411.2) | -5 (54.4) |
| Visit 5 | 97 | 1450.5 (391.8) | 1447.4 (375.2) | 3.1 (62.7) |
| Visit 6 | 84 | 1526.1 (374.0) | 1527.2 (342.7) | -1.1 (74.4) |
| Visit 7 | 61 | 1596.5 (331.6) | 1593.5 (304.3) | 2.9 (72.7) |
| PBMAS males |  |  |  |  |
| Visit 1 | 107 | 1091.9 (502.3) | 1079.9 (485.3) | 11.9 (80.5) |
| Visit 2 | 105 | 1195 (505.6) | 1202.5 (506.6) | -7.4 (61.7) |
| Visit 3 | 103 | 1420.1 (552.0) | 1417.9 (547.5) | 2.2 (65.9) |
| Visit 4 | 86 | 1595.7 (588.6) | 1603.2 (577.6) | -7.6 (73.2) |
| Visit 5 | 83 | 1794.8 (559.2) | 1806 (548.8) | -11.2 (86.6) |
| Visit 6 | 69 | 2000.6 (535.7) | 1997.7 (519.2) | 2.8 (97.6) |
| Visit 7 | 51 | 2104.5 (525.7) | 2094.4 (488.3) | 10.1 (115.7) |

Data are means (SD). For brevity, results for the first 7 visits only are presented for PBMAS

**Additional file 2f** Variance explained by selected SITAR models**:** residual variances compared to the corresponding fixed effects model (i.e., treating data as cross-sectional).

|  | ALSPAC | BMDCS | PBMAS |
| --- | --- | --- | --- |
| Females | 90.1% | 97.6% | 96.2% |
| Males | 94.5% | 96.9% | 95.9% |
