# Supplementary material for: Using linear and natural cubic splines, SITAR, and latent trajectory models to characterise nonlinear longitudinal growth trajectories in cohort studies
Source: BMC Med Res Methodol. 2022 Mar 15;22:68. doi: 10.1186/s12874-022-01542-8 (PMC8925070; doi:10.1186/s12874-022-01542-8)
Supplement: Supplementary file 3 — Additional file 3. Fit statistics and predicted mean BMC latent trajectories for growth mixture models with 1 to 5 latent classes. [file 12874_2022_1542_MOESM3_ESM.docx]

# **Additional file 3** Fit statistics and predicted mean BMC latent trajectories for growth mixture models with 1 to 5 latent classes

**Additional file 3a** Fit statistics for growth mixture models with with 1 to 5 latent classes

| Cohort | Number of classes | npm | BIC | Entropy | % in each class |
| --- | --- | --- | --- | --- | --- |
| ALSPAC Females | 1-class | 9 | 211303.8 | - | 100 |
|  | 2-class | 16 | 208953.8 | 0.65 | 70.7, 29.3 |
|  | 3-class | 23 | 207534.9 | 0.65 | 58.3, 28.4, 13.3 |
|  | 4-class | 30 | 206783.4 | 0.63 | 38.2, 30.8, 20.8, 10.3 |
|  | 5-class | - | - | - | - |
|  |  |  |  |  |  |
| ALSPAC Males | 1-class | 12 | 191801.2 | - | 100 |
|  | 2-class | 22 | 188187.5 | 0.60 | 65.6, 34.4 |
|  | 3-class | 32 | 187032.4 | 0.56 | 53.9, 24.0, 22.2 |
|  | 4-class | 42 | 186317.5 | 0.56 | 37.8, 24.5, 22.8, 14.8 |
|  | 5-class | 52 | 185747.9 | 0.59 | 34.3, 25.3, 19.5, 15.5, 5.4 |
|  |  |  |  |  |  |
| BMDCS Females | 1-class | 11 | 30495.27 | - | 100 |
|  | 2-class | 20 | 29808.35 | 0.55 | 59.8, 40.2 |
|  | 3-class | 29 | 29575.34 | 0.58 | 60.2, 20.7, 19.1 |
|  | 4-class | 38 | 29451.66 | 0.64 | 56.4, 24.4, 13.7, 5.5 |
|  | 5-class | 47 | 29371.83 | 0.65 | 31.4, 31.4, 22.1, 9.6, 5.5 |
|  |  |  |  |  |  |
| BMDCS Males | 1-class | 11 | 30338.56 | - | 100 |
|  | 2-class | 20 | 29623.73 | 0.60 | 62.2, 37.8 |
|  | 3-class | 29 | 29374.76 | 0.57 | 58.9, 23.4, 17.6 |
|  | 4-class | 38 | 29255.09 | 0.61 | 50.1, 24.3, 18.3, 7.3 |
|  | 5-class | 47 | 29185.02 | 0.60 | 46.5, 25.2, 13.3, 7.5, 7.5 |
|  |  |  |  |  |  |
| PBMAS Females | 1-class | 12 | 13675.06 | - | 100 |
|  | 2-class | 22 | 13502.25 | 0.71 | 60.6, 39.4 |
|  | 3-class | 32 | 13390.31 | 0.75 | 44.9, 37.0, 18.1 |
|  | 4-class | 42 | 13323.66 | 0.76 | 39.4, 32.3, 15.0, 13.4 |
|  | 5-class | 52 | 13293.41 | 0.79 | 37.8, 29.9, 15.0, 12.6, 4.7 |
|  |  |  |  |  |  |
| PBMAS Males | 1-class | 12 | 11943.44 | - | 100 |
|  | 2-class | 22 | 11694.18 | 0.77 | 58.9, 41.1 |
|  | 3-class | 32 | 11631.61 | 0.72 | 37.5, 35.7, 26.8 |
|  | 4-class | 42 | 11588.09 | 0.78 | 32.1, 23.2, 22.3, 22.3 |
|  | 5-class | 52 | 11646.34 | 0.79 | 30.4, 20.5, 17.0, 17.0, 15.2 |

npm = number of parameters

**Additional file 3b** predicted latent trajectories for growth mixture models with 1 to 4 latent classes: ALSPAC Females

**Additional file 3c** predicted latent trajectories for growth mixture models with 1 to 5 latent classes: ALSPAC Males

**Additional file 3d** predicted latent trajectories for growth mixture models with 1 to 5 latent classes: BMDCS Females

**Additional file 3e** predicted latent trajectories for growth mixture models with 1 to 5 latent classes: BMDCS Males

**Additional file 3f** predicted latent trajectories for growth mixture models with 1 to 5 latent classes: PBMAS Females

**Additional file 3g** predicted latent trajectories for growth mixture models with 1 to 5 latent classes: PBMAS Males

**Additional file 3h** Posterior classification table showing the mean posterior probabilities of belonging to each latent class among individuals *a posteriori* classified to the given class from the selected growth mixture models.

|  | Mean of the posterior probabilities of belonging to each class | | | |
| --- | --- | --- | --- | --- |
|  | Class 1 | Class 2 | Class 3 | Class 4 |
| ALSPAC Females |  |  |  |  |
| Class 1 (n=2337) | 0.86 | 0.04 | 0.11 | - |
| Class 2 (n=531) | 0.05 | 0.86 | 0.10 | - |
| Class 3 (n=1139) | 0.12 | 0.09 | 0.79 | - |
|  |  |  |  |  |
| ALSPAC Males |  |  |  |  |
| Class 1 (n=1339) | 0.88 | 0.12 | - | - |
| Class 2 (n=2549) | 0.13 | 0.87 | - | - |
|  |  |  |  |  |
| BMDCS Females |  |  |  |  |
| Class 1 (n=101) | 0.91 | 0.00 | 0.09 | - |
| Class 2 (n=93) | 0.04 | 0.87 | 0.09 | - |
| Class 3 (n=294) | 0.18 | 0.11 | 0.71 | - |
|  |  |  |  |  |
| BMDCS Males |  |  |  |  |
| Class 1 (n=176) | 0.91 | 0.09 | - | - |
| Class 2 (n=289) | 0.18 | 0.82 | - | - |
|  |  |  |  |  |
| PBMAS Females |  |  |  |  |
| Class 1 (n=50) | 0.86 | 0.04 | 0.09 | 0.01 |
| Class 2 (n=19) | 0.06 | 0.92 | 0.00 | 0.00 |
| Class 3 (n=41) | 0.08 | 0.03 | 0.83 | 0.06 |
| Class 4 (n=17) | 0.01 | 0.02 | 0.07 | 0.90 |
|  |  |  |  |  |
| PBMAS Males |  |  |  |  |
| Class 1 (n=42) | 0.81 | 0.08 | 0.11 | - |
| Class 2 (n=40) | 0.03 | 0.94 | 0.03 | - |
| Class 3 (n=30) | 0.11 | 0.07 | 0.82 | - |

A model has a good discrimination capacity if the diagonal terms are close to 1 and all other terms are close to 0.
